# Biomarkers for the Molecular Diagnosis of IgE-Mediated Hymenoptera Venom Allergy in Clinical Practice

**DOI:** 10.3390/ijms26010270

**Published:** 2024-12-31

**Authors:** Florin-Dan Popescu, Mariana Preda, Darío Antolín-Amérigo, Natalia Rodríguez-Otero, Elena Ramírez-Mateo, Sylwia Smolinska

**Affiliations:** 1 Faculty of Medicine, Department of Allergology Nicolae Malaxa Clinical Hospital, Carol Davila University of Medicine and Pharmacy, 022441 Bucharest, Romania; florindanpopescu@allergist.com (F.-D.P.); mariana.preda@umfcd.ro (M.P.); 2 Instituto Ramón y Cajal de Investigación Sanitaria (IRYCIS), Ramón y Cajal University Hospital, 28034 Madrid, Spain; dario.antolin2@gmail.com (D.A.-A.); nataliarot23@gmail.com (N.R.-O.); elenaramirezhryc@gmail.com (E.R.-M.); 3 Faculty of Medicine, Department of Clinical Immunology, Wroclaw Medical University, 51-616 Wroclaw, Poland

**Keywords:** biomarkers, molecular diagnosis, Hymenoptera venom allergy

## Abstract

Hymenoptera venom allergy (HVA) is a potentially life-threatening condition, making accurate diagnosis crucial for identifying significant IgE sensitizations and enabling effective venom immunotherapy. In this review, we provide a detailed overview of biomarkers for the molecular diagnosis of IgE-mediated hypersensitivity to Hymenoptera insect venoms in clinical practice, and we present, in a structured manner, their importance in differentiating genuine sensitizations versus cross-sensitizations using different diagnostic procedures. Updated algorithms are provided, along with the advantages and limitations of molecular diagnosis approaches. Geographical variations and rare species may pose further challenges in diagnosing and treating HVA, adding complexity to HVA management. This review informs readers about performing tailored diagnostics based on molecular allergen biomarkers and subsequent treatment strategies.

## 1. Introduction

IgE-mediated HVA is a disorder characterized by potentially life-threatening immediate allergic reactions to the venom of stinging insects from the Hymenoptera order, which includes bees, wasps, yellow jackets, hornets, and ants. Because venom immunotherapy (VIT) offers a curative treatment for most patients, by modifying their specific immune responses, an accurate diagnosis is crucial for identifying relevant IgE sensitization and enabling adequate specific treatment. This may include, after detailed history taking, in vitro IgE immunoassays and basophil activation tests using molecular allergen biomarkers, usually subsequently to in vivo allergy tests and/or in vitro measurement of serum specific IgE (ssIgE) against whole-venom extracts (wve-s) [1,2,3,4,5].

Hymenoptera stings may cause large local reactions (LLRs) and anaphylaxis in adults, accounting for nearly half of all cases in this category and about one-fifth in children. In Europe, HVA is recognized as leading cause of anaphylaxis in adults. It is estimated that between 60% and 95% of people have experienced at least one Hymenoptera sting in their lifetime. LLRs occur in 2.5% to 25% of individuals, whereas systemic sting reactions (SSRs) are relatively rare, affecting only 0.3% to 9% of adults [1,6,7,8,9].

The prevalence of ssIgE to wve-s in the general adult population is significantly higher than clinical reactions. There is a disparity between the IgE sensitization rates and their clinical relevance. Approximately 9% to 42% of the adult population show a sensitization to Hymenoptera venom without a previous history of a sting reaction. Most sensitized individuals will not experience a clinically significant reaction after being stung [10,11,12,13]. This phenomenon can be attributed to several factors, including IgE sialylation, non-IgE blocking antibodies, intracellular signaling pathways, and regulatory factors. Additionally, venom-specific IgE may enhance natural defense mechanisms by increasing mast cell responses and facilitating venom neutralization by releasing enzymes. However, the possibility of an allergic reaction to a future sting cannot be excluded entirely. Currently, there are no clear guidelines for managing such cases effectively [14,15].

The clinical manifestations of adverse reactions can vary significantly, ranging from LRRs to potentially life-threatening SSRs. Up to 75% of subjects with severe anaphylaxis after Hymenoptera insect sting risk further severe reactions if re-stung. Risk factors and cofactors potentially associated with severe or fatal insect sting-induced anaphylaxis include older age, male sex, hereditary α-tryptasemia, mast cell disorders, cardiovascular diseases, and antihypertensive drugs, such as intake of beta-blockers and angiotensin-converting enzyme inhibitors in temporal proximity to allergen exposure. Patients with HVA, especially those with severe anaphylaxis, frequently have concomitant clonal mast cell disease in the form of systemic mastocytosis or monoclonal mast cell activation syndrome. The prevalence of mast cell disorders in HVA patients is significantly higher (up to 8%) than in the general population, with nearly 30% of mastocytosis patients experiencing anaphylaxis related to HVA, which typically presents with cardiovascular symptoms rather than urticaria or angioedema [16,17,18,19,20]. 

Hymenoptera insect stings are generally well tolerated and usually cause limited local reactions, characterized by self-limiting erythema and oedema associated with pain, but they can also induce hypersensitivity reactions mediated by IgE antibodies specific to venom components. Moreover, serum sickness-type reactions and unusual reactions have been reported, including toxic ones after unusual massive envenomation [12,21,22,23].

The first step in the diagnosis of IgE-mediated HVA is represented by routine in vivo and in vitro tests using wve-s: skin prick testing with wve-s (SPT-wve), intradermal testing with wve-s (IDT-wve), and/or determination of ssIgE to wve-s. As a second-level evaluation, serologic testing using molecular venom allergens can further discriminate genuine IgE sensitization and interspecies cross-reactivity. Particular allergen molecules can serve as biomarkers for a primary, genuine or family-/species-specific sensitization, when used as recombinant forms, such as honey bee venom phospholipase A2, acid phosphatase, melittin, icarapin, and vespid venom phospholipases A1 and antigens 5. Many important native Hymenoptera venom allergen components are glycosylated and carry cross-reactive carbohydrate determinants (CCDs), such as honey bee venom phospholipase A2, acid phosphatase, dipeptidyl peptidase IV, icarapin, and Hymenoptera venom hyaluronidases. In the case of double-positive ssIgE detection for bee and vespid wve-s, there are different explanations, such as genuine double sensitization to species-specific proteins in both venoms, clinically irrelevant positive results due to specific IgE against CCDs or cross-reactivity due to IgE sensitization to protein epitopes expressed by homologous proteins from both venoms. The patient’s serum screening for the detection of ssIgE to CCDs is usually performed using CCD-rich substrates such as nMUXF3 (CCD component from pineapple bromelain). Determination of ssIgE to CCD MUXF3, in contrast to multiple sensitizations to pollen or plant foods, is of limited use. As CCDs differ in insect venoms, the sensitivity of the nMUXF3 assay is relatively low. The presence of ssIgE to CCDs does not exclude genuine double sensitization. Modern IgE immunoassays use recombinant allergens, produced in various expression systems to be CCD-free [3,9,24].

## 2. Molecular Insect Venom Allergens Used as Biomarkers for the Diagnosis of IgE-Mediated HVA in Clinical Practice

The Hymenoptera order includes many different species of flying and non-flying insects. Representatives of the Apidae (bees), Vespidae (wasps, yellow jackets, and hornets), and Formicidae (stinging ants) families capable of injecting venom into their prey or as a defense mechanism, use completely modified ovipositors into stinging apparatus at the terminal end of their abdomen. Honeybees have barbed stingers, which often remain attached to the skin after a single sting, while social wasps do not have a barbed stinger and can sting multiple times. Bee and wasp venoms contain low-molecular-weight compounds responsible for local inflammatory reactions, such as vasoactive amines like histamine, noradrenaline, dopamine, and serotonin; bee venom peptides like neurotoxin apamin, cytotoxic and antimicrobial melittin, and mast cell degranulating peptide; wasp venom peptides like cytotoxic and antimicrobial mastoparan, and vasoactive kinin; and high-molecular-weight proteins, such as enzymes: phospholipases, hyaluronidases, acid phosphatases, dipeptidyl peptidases; and other proteins with unknown functions, like vitellogenins and wasp venom antigen 5 molecules. All these high-molecular-weight compounds act as allergens involved in IgE-mediated SSRs. It is estimated that the European honeybee delivers 50–147 µg of venom/sting, a bumblebee 10–31 µg of venom/sting, a yellow jacket 1.7–3.1 µg of venom/sting, a paper wasp 4.2–17 µg of venom/sting, while a hornet has a venom dry weight poison sac of 260 µg. It is considered that a dose of 100 μg venom for subcutaneous administration in VIT is equivalent to the dry weight of approximately two bee stings or five wasp stings [5,24,25,26,27,28].

The Formicidae family includes all ants, most of which can bite with pincer-shaped mandibles; however, only some ants with brief winged reproductive stages in their life cycle have also developed the ability to sting with abdominal stingers. Fire ant venom is primarily made of cytotoxic and antimicrobial piperidine alkaloids known as solenopsins, causing sterile pustules associated with their stings, but also contains protein allergens, including venom antigen 5 molecules. It was assessed that a red imported fire ant delivers 0.6 µg venom alkaloids and 10–100 ng of protein per sting [24,26,29].

The most prominent honeybee species known to elicit HVA worldwide is *Apis mellifera*. Beekeepers and rural populations are at higher risk of developing honeybee venom allergy. Along with certain vespids, especially *Vespula vulgaris* and *Vespula germanica*, they are the most common elicitors of clinically significant sting reactions. Generally, stings by *Polistes* spp., *Dolichovespula* spp., and *Vespa* spp. are less frequent. In addition, stings from bumblebees are usually very rare but common in horticulture if these insects are used for pollination in greenhouses. From the Vespinae subfamily, *Vespula* spp. are prominent as allergy-eliciting species, mainly in the Northern Hemisphere. *Dolichovespula* spp. are important species in North America and Europe but live farther from humans. From the Polistinae subfamily, which has a worldwide distribution, *Polistes* spp. have greater significance in warmer areas in the United States and Mediterranean regions of Europe. In South America, *Polybia* spp. are of particular importance. Additionally, allergies to *Vespa* spp. are also reported in Europe due to *Vespa crabro* and may increase in frequency due to the spread of invasive species such as *Vespa velutina nigrithorax* [3,4,9,30,31].

Other stings caused by fire ants, needle ants, and jumper ants are also involved in HVA in endemic regions typically outside Europe. The *Solenopsis* spp. fire ants, native to South America, are found in Southern and Southeast US, Mexico, Australia, New Zealand, and several Caribbean and Asian countries where they are often referred to as “imported fire ants”. The Asian needle ant has spread from Far Eastern Asia to New Zealand and North America, while jumper ants are most frequently found in Tasmania and Australia. Invasive alien ant species are also detected in Southern Europe, such as red imported fire ants as a mature population in Sicily, Asian needle ants in Naples and near Lake Como in northern Italy. Accidental exposure with anaphylaxis to red fire ants when handling infested wood from South America was also reported in Málaga, Spain [32,33,34,35,36].

Hymenoptera venom extracts are traditionally obtained from collected insect workers as electrostimulated venom (ESV) or capillary-extracted venom (CEV). ESV is obtained by electrical stimulation of bees (“electric milking”) directly on the field in front of the hives. Mild, high-frequency electric shocks cause them to sting through a nylon net and deposit a droplet of glandular venom (GV) onto a glass plate. CEV is collected directly from the bee or wasp sting by gently squeezing the dissected hymenopteran venom glands, including the reservoir (“reservoir disrupting”). Both extracts are diluted in water for injection, filtered, quantified, aliquoted, lyophilized and frozen until used. Another method for extracting fire ant venom is insect stress caused by immersion in a dual-phase mixture of apolar organic solvent and water [21,37,38,39,40].

The honeybee *Apis mellifera* venom (AmV) consists of a complex mixture of allergenic compounds (Table 1), among which phospholipase A2 (calcium-dependent hydrolase) Api m 1, hyaluronidase (glycosyl hydrolase) Api m 2, acid phosphatase Api m 3, melittin Api m 4, dipeptidyl peptidase IV (serine-peptidase) Api m 5, and icarapin Api m 10 represent allergen biomarkers currently used in IgE immunoassays as CCD-free molecules [4,9,41].

Allergen venom biomarkers are presented after the official allergen nomenclature for single allergens based on the abbreviated Latin name of the venom source (the first three letters of the genus), the first letter of the species name, and the number of the allergen usually in the sequence of their discovery.

Api m 1 is a glycoprotein of the phospholipase 2 (PLA2) family, secreted into the *Apis mellifera*’s venom sac, contributing 12–16% of venom dry weight. Its production exhibits seasonal variations, similar to mellitin. Following activation by melittin, Api m 1 targets cellular, bacterial, or surfactant phospholipids. Api m 1 exerts toxic effects independent of its allergenic potency. PLA2 venom allergens are present in honeybees and bumblebees, but not in vespids’ venom. Api m 1 displays an amino acid sequence similarity of 50% or higher with other bee PLA2, such as Api c 1 and Api d 1 from Asiatic honeybees. Instead, PLA1 allergens in wasp venoms do not share sequence identity or structural similarity with Api m 1. The rApi m 1 is an allergen biomarker for genuine AmV sensitization and allows discrimination between AmV and *Vespula*/*Polistes* spp. venom sensitization but does not allow differentiation between AmV and bumblebee venom. The prevalence of IgE sensitization to individual AmV allergens, including Api m 1, in AmV-allergic subjects varies depending on several factors such as geographic location, single or double positivity to AmV and vespid venoms, immunoassay format, and the use of recombinant allergens from bacterial or insect cell expression systems or natural purified allergen components. Of the identified AmV allergens, Api m 1 is considered the most prominent one in terms of the prevalence of sensitization, levels of ssIgE, and quantitative correlation between Api m 1-ssIgE and AmV-ssIgE. Although the diagnostic sensitivity of ssIgE to rApi m 1 is not high, ranging from 57% to 62%, its very high diagnostic specificity of 97% to 100% makes rApi m 1 a relevant allergen biomarker. In AmV-allergic patients with a lower prevalence of ssIgE to rApi m 1, using additional AmV allergens such as Api m 3, Api m 4, and Api m 10 increases the chance of indicating genuine sensitization to AmV [3,4,9,41,42].

Api m 2, also known as honeybee hyaluronidase, is a secreted glycoprotein which contributes to 2% of venom dry weight, facilitates the penetration of other venom constituents across the extracellular matrix adjacent to the sting area, and releases shorter fragments of hyaluronan with pro-inflammatory effects [42,43,44].

Cross-reactivity between homologous native hyaluronidases Api m 2 and Ves v 2 can result in apparent double-positivity to AmV and vespid wve-s, mainly due to CCDs. Despite an amino acid sequence similarity of 50% or higher between Api m 2 and other Hymenoptera venom hyaluronidases, rApi m 2 seldom cross-reacts with its vespid counterparts. Therefore, rApi m 2 is a helpful biomarker for AmV sensitization due to limited cross-reactivity with wasp hyaluronidases without CCDs. However, cross-reactivity with these minor allergens cannot be entirely excluded. Although Api m 2 is considered a significant allergen in AmV, the corresponding hyaluronidases in VvV (Ves v 2) and PdV (Pol d 2) are considered less relevant in vespid venom allergies. Results should be interpreted with care in the context of clinical history. The diagnostic specificity of ssIgE to rApi m 2 for AmV allergy is high, 90% to 100% [4,41,44,45,46].

Api m 3 is a glycoprotein of the acid phosphatase family possibly involved in catalyzing the release of purines, mainly adenosine, which act as multitoxins [24]. Api m 3 is secreted into the venom sac where it contributes about 1.5–2% of venom dry weight, and is a major specific allergen of AmV, with a prevalence of up to 63% of ssIgE in AmV-allergic patients. rApi m 3 represents a biomarker for genuine sensitization to AmV and allows discrimination between AmV and *Vespula*/*Polistes* spp. venom IgE sensitization. It is also a valuable allergen marker in diagnosing AmV allergy in Api m 1-negative patients. Moreover, Api m 3 sensitization is not detected in subjects without AmV allergy, even in those with a history of bee stings or detectable ssIgE to AmV wve. It has also been suggested that Api m 3 might be underrepresented in therapeutic AmV wve-s for VIT, which may affect the outcome of VIT if it is the dominant sensitizer [4,47,48,49,50].

Api m 4, represented by melittin, is the main component of AmV, representing about half of venom dry weight, and together with Api m 1, it accounts for more than 60% of it. This bee venom peptide is the main toxin with haemolytic, cardiotoxic, and antimicrobial properties. It is responsible for the local inflammation and pain sensation at the bee sting site. Api m 5 catalyzes the conversion from promelittin to melittin only in the venom sac, thus protecting the bees from its cytotoxic effects. Api m 4 has much lower allergenic properties than Api m 1 or Api m 2. It is considered a minor allergen, and the sera of 25–50% of patients allergic to AmV containing ssIgE are against Api m 4. Synthetic Api m 4 (sApi m 4) is an allergen biomarker for bee venom IgE sensitization, and it allows discrimination between AmV and vespid venom IgE sensitization. Furthermore, sApi m 4 is a putative allergen biomarker for increased risk of systemic reactions during the initiation phase of VIT and for more severe systemic reactions after a bee sting [4,40,41,47,50].

Api m 5, also known as dipeptidyl peptidase IV or allergen C, is another major allergen recognized by ssIgE in most AmV-allergic patients. Although it does not cross-react with other major bee venom allergens, including Api m 1, Api m 2, Api m 3, and Api m 4, this enzyme is the second most common cause of allergenic cross-reactivity between AmV and common wasp venoms after hyaluronidase. The high cross-reactivity of Api m 5 with dipeptidyl peptidases Ves v 3 and Pol d 3 prevents its widespread use as an allergen biomarker for AmV sensitization. Using rApi m 5 in immunoassays remains a diagnostic option in cases where bee venom allergy is highly likely and ssIgE antibodies against other molecular allergens are not found. Moreover, detecting ssIgE to rApi m 5 does not exclude primary common wasp venom sensitization [4,9,41,47,51].

Api m 10, also known as icarapin, is a glycoprotein with unknown biological function secreted into the venom sac of honeybees, contributing 0.8% of venom dry weight. Icarapine is a term created from the Greek mythology name Icarus and the genus name *Apis*, thus indicating its unstable nature and rapid degradation. Api m 10 is a major allergen of AmV, with the prevalence of ssIgE against rApi m 10 being up to 75% in AmV-allergic patients. It is a biomarker for genuine AmV sensitization, as it does not cross-react with allergens from other Hymenoptera venoms, and it allows discrimination between AmV and *Vespula*/*Polistes* spp. venom IgE sensitization. Moreover, it is particularly valuable in Api m 1-negative patients. Api m 10 is unstable in both native and recombinant forms, which may explain its underrepresentation in therapeutic AmV wve-s. Therefore, the sensitization to this allergen has been associated with poorer responses to, or even therapeutic failure of AmV VIT, especially in patients with dominant sensitization (levels of ssIgE to rApi m 10 greater than 50% of those of ssIgE to AmV wve-s). Conversely, lower levels of ssIgE against rApi m 10 were reported in patients experiencing severe adverse effects during VIT, although they cannot distinguish between severe versus non-severe systemic reactions to honeybee stings [9,46,52,53,54].

The common wasp/yellow jacket *Vespula vulgaris* venom (VvV) consists of a complex mixture of allergenic compounds (Table 2), among which phospholipase A1 Ves v 1 and venom antigen 5 molecule Ves v 5 represent the allergen biomarkers currently used in IgE immunoassays as CCD-free molecules. Ves v 1 and Ves v 5 are marker allergens for VvV sensitization, allowing discrimination between VvV and AmV sensitization, but their high cross-reactivity with Pol d 1 and Pol d 5, respectively, limits their use as an allergen marker to clearly discriminate between VvV and PdV sensitizations [9,41].

The prevalence of sensitization to VvV components, including Ves v 1 and Ves v 5, in VvV-allergic patients varies, depending on multiple factors, such as location, single or double positivity to VvV and other Hymenoptera venoms, immunoassay format, the use of recombinant versus native purified allergens, and positivity cut-off values [13,55,56,57].

Ves v 1 is a phospholipase A1 (PLA1) secreted into the venom sac of *Vespula vulgaris*, contributing to 6–14% of the VvV dry weight. The diagnostic sensitivity of ssIgE to this allergen biomarker ranges widely to up to 80% in VvV-allergic patients. rVes v 1 is considered a marker allergen for genuine VvV sensitization exhibiting a diagnostic specificity of 94–100% in this case. Thus, detecting ssIgE to rVes v 1 confirms genuine sensitization to VvV and helps identify the primary sensitizer in patients with double IgE positivity to AmV and VvV wve-s. Ves v 1 has high cross-reactivity with Pol d 1, preventing accurate discrimination between VvV and PdV sensitizations. It was suggested that the relative amount of ssIgE to distinct venom PLA1 allergens or IgE-inhibition assays may help identify the primary venom sensitizer [4,56,58].

Ves v 5, also known as VvV antigen 5, is a member of the CAP (Cysteine-rich secretory proteins/CRISPs, antigen 5/Ag5, and pathogenesis-related PR-1) superfamily proteins found in a remarkable range of venomous animal species. rVes v 5 is a biomarker for genuine VvV sensitization and a major allergen in VvV-allergic patients, with Ves v 5-ssIgE being revealed in 82–98% of VvV allergic patients. Nearly all patients who are primarily sensitized to VvV have ssIgE to Ves v 5 and/or Ves v 1, while a patient without detectable ssIgE to both Ves v 5 and Ves v 1 is unlikely to have primary VvV sensitization. Because AmV lacks Ves v 5 homologues, rVes v 5 discriminates AmV sensitization in patients who are double-positive to VvV and AmV wve-s. Asymptomatic Ves v 5 sensitization is frequent, with up to 20% of the general population [4,13,57,58,59].

Ag5 allergens of different Vespinae representatives, including *Vespula*, *Dolichovespula* and *Vespa* spp., display pronounced cross-reactivity. This hampers its use for the differential diagnosis of VvV versus PdV genuine sensitization. However, the relative amount of ssIgE to distinct Ag5 allergen components may help identify the primary venom sensitizer. In patients with suggestive clinical history and proportion between ssIgE against rVes v 5 and rPol d 5 greater than 2 and IgE-inhibition assay may help perform better differential diagnosis between VvV and PdV sensitization [4,60,61].

Because non-Hymenoptera insect bites can also cause allergic reactions in humans, their salivary allergens were assessed. Among them, Tab y 5 from *Tabanus yao* horsefly was mentioned as an Ag5-related protein similar to Ves v 5 and Vesp ma 5 from *Vespula vulgaris* and *Vespa magnifica*, respectively. It is possibly involved in the wasp-horsefly syndrome in which wasp venom sensitization was revealed in cases of anaphylaxis to *Tabanus* spp. bites. This may be explained by protein sequence homology, but co-sensitisation should also be considered because individuals who spend more time outdoors in rural areas are more likely to be exposed to both types of insects. Notably, many tabanids may resemble wasps to untrained observers, and this must be considered when evaluating patients with anaphylaxis due to unidentified flying insects [62,63,64].

The paper wasp *Polistes dominula* venom (PdV) consists of a complex mixture of allergenic compounds (Table 3), among which phospholipase A1 Pol d 1 and venom Ag5 molecule Pol d 5 represent a significant portion of the dry PdV weight and allergen biomarkers used in IgE immunoassays as CCD-free biomarkers. Pol d 1 and Pol d 5 are marker allergens for PdV sensitization, allowing discrimination between PdV and AmV sensitization, but their high cross-reactivity with Ves v 1 and Ves v 5, respectively, limits their use as a marker to clearly discriminate between PdV and VvV sensitizations [9,41].

Pol d 1 sensitization is most frequent in PdV-sensitized Italian patients, ranging from 97% to 100% in those with concomitant VvV sensitization and those mono-sensitized to PdV, respectively. This major allergen can distinguish *Polistes* primary sensitizations with good diagnostic accuracy, which supports its use in clinical practice. It is frequently involved in cases of positivity to a single PdV molecule (48% in double- and 80% in mono-sensitized patients) [65].

Ag5 molecules are the most potent allergens in vespid venoms and are found in nearly all Vespoidea species, with varying sequence homology. Pol d 5 displays structure identity higher than 80% with Polistinae homologues and around 60% with *Vespula* and *Vespa* spp. ones. rPol d 5 cross-reactivity within the homologues Ag5 proteins hampers its use for the differential diagnosis of PdV versus VvV or other vespid venom genuine sensitization. For example, many Spanish PdV-allergic patients with Pol d 5-ssIgE exhibit sensitization to Ves v 5, but also to Poly s 5 from the South American wasp *Polybia scutellaris* and Pol a 5 from North American paper wasp *Polistes annularis*. Moreover, many VvV-allergic patients from Germany, where PdV primary sensitization is believed to be absent, reveal detectable Pol d 5-ssIgE. rPol d 5 is an allergen biomarker for genuine vespid venom sensitization, most suitable in regions with a high prevalence of *Polistes dominula* exposure and allergy, such as warmer climates or Mediterranean regions, which helps to identify the PdV as the primary sensitizer in cases with double IgE sensitization against AmV and PdV wve-s. Because diagnostic sensitivity of ssIgE against Pol d 5 varies widely between 20% and 80% in PdV-allergic patients, genuine PdV or other vespid venom sensitization cannot be excluded in a patient without detectable Pol d 5-ssIgE. The prevalence of sensitization to PdV allergen components, including Pol d 5, in PdV-allergic patients varies, depending on multiple factors such as geography, single or double positivity to PdV and other Hymenoptera venoms, immunoassay format, the use of recombinant or natural purified allergens, and positivity cut-off values. Noteworthy, Pol d 5 sensitization in the general population is not synonymous with PdV allergy due to the high prevalence of asymptomatic Vespid sensitization [4,65,66,67,68].

In selected cases, such as those with Pol d 5-ssIgE levels at least twice those of Ves v 5-ssIgE, primary PdV sensitization is probable. Better diagnostic accuracy for PdV sensitization may be obtained when receiver operator curves are built using combined IgE ratios of Pol d 5/Ves v 5, Pol d 1/Ves v 1, and PdV and VvV wve-s. Moreover, IgE-inhibition assays using PdV and VvV wve-s or molecular markers Pol d 5 and Ves v 5 are additionally helpful [61,68,69,70,71].

Although molecular allergen biomarkers are identified in the venoms of various stinging ants (Table 4), such as the red imported fire ant phospholipase A1B (Sol i 1) which is cross-reactive with Ves v 1, the fire ant venom antigen 5 homologous molecules (Sol i 3 and Sol r 3) and Asian needle ant Pac c 3 which is cross-reactive with Ves v 5, and the Australian jumper ant’s highly basic, low-molecular-weight unique peptide pilosulin 3, these are not available yet for commercial IgE immunoassays [36,72,73].

Finally, it is crucial to emphasize that in Hymenoptera venom IgE immunoassays, the quantitative results of ssIgE to a molecular allergen or wve-s are neither predictive of nor correlated with the severity of an allergic sting reaction [4].

## 3. IgE Immunoassays Using Biomarkers for the Molecular Diagnosis of IgE-Mediated HVA in Clinical Practice

Modern IgE immunoassays are essential in vitro tools for measuring ssIgE antibodies against natural insect wve-s and molecular venom allergen components, especially recombinant or synthetic molecules. They can be used to measure IgE-mediated sensitization to Hymenoptera venoms. Standard reference commercial singleplex in vitro methods for ssIgE to individual natural venom extracts and molecular venom components use either solid-phase coupled allergens (i.e., fluorescence enzyme immunoassay) or liquid-phase allergens (i.e., chemiluminescence immunoassay) [74,75].

The fluorescence enzyme immunoassay (FEIA) with capsulated cellulose polymer solid-phase coupled allergens (ImmunoCAP^®^, Thermo Fisher Scientific Inc., Phadia AB, Uppsala, Sweden) is usually used as a singleplex assay to measure ssIgE to Hymenoptera venom allergens (Table 5). Allergens are covalently coupled in FEIA to the hydrophilic carrier polymer consisting of a cyanogen bromide-activated cellulose derivative with a large surface for protein binding. This method uses β-galactosidase-labeled anti-IgE monoclonal antibodies and 4-methylumbelliferyl-β-galactoside as a fluorogenic substrate, with the fluorescence measurement being performed with a fluorocounter [9,74,76,77].

The enzyme-enhanced chemiluminescence immunoassay (CLIA) with liquid-phase allergens is another advanced singleplex detection method for ssIgE that exploits liquid-phase kinetics in a bead format (3gAllergy™ Immulite^®^ 2000 and Immulite^®^ 2000 XPi immunoassay; Siemens Healthcare Diagnostics Inc., Erlangen, Germany). Insect venom allergens (Table 5) covalently bound to soluble biotinylated polylysine polymer in a fluid phase bind to streptavidin-coated polystyrene bead in the reaction tube (through a streptavidin−biotin interaction). This method utilizes alkaline phosphatase enzyme-labelled anti-IgE monoclonal antibodies and adamantyl 1,2-dioxetane aryl phosphate as a chemiluminescent substrate, with the chemiluminescence being measured using a luminometer [9,74,76,78,79].

Another chemiluminescence immunoassay (Noveos^®^, Hycor Biomedical, Garden Grove, CA, USA) assesses only ssIgE against CCD-free Ves v 1 and Ves v 5 using biotinylated soluble allergens coupled with streptavidin-coated magnetic beads. Another reliable singleplex immunoassay, the reversed enzyme allergosorbent test (REAST) with liquid-phase allergens (Allerg-O-Liq™, Dr. Fooke-Achterrath Laboratorien GmbH, Neuss, Germany) using microwells and based on a sandwich enzyme-linked immunosorbent assay (ELISA), may also be used for the determination of ssIgE antibodies against additional CCD-free honey bee venom allergens, Api m 1, Api m 2, and Api m 10, besides Ves v 1 and Ves v 5. A novel rapid allergy lateral flow assay (ALFA™, Dr. Fooke-Achterrath Laboratorien GmbH, Neuss, Germany) for the detection of ssIgE to the above-mentioned bee and wasp venom CCD-free allergen components utilizes the capillary flow principle, biotinylated liquid insect venom allergens applied in a test cassette, antibodies coupled to gold particles, and evaluation of the colorimetric reaction at the test line [9,74,76,80].

The IgE-inhibition using FEIA was not reported with hymenopteran venom molecular allergens in clinical practice, but reciprocal IgE-inhibition assays, in which the patient’s serum is incubated separately with different wve preparations at increasing serial dilutions and, subsequently, the mixtures are used as samples in the FEIA immunoassay, may be used in cases of double positivity to bee and wasp venoms. The extents of homologous (blockage of venom-specific IgE by the same venom) and heterologous (blockage of the venom-specific IgE by the other venom) inhibition are computed with the following formula: % inhibition = 100 − [IgE inhibited sample (kU/L) × 100/IgE anti-venom (kU/L) at zero concentration of venom]. Values above 85% are considered completely cross-reactive for bee and wasp venoms in general, and a percentage of heterologous inhibition of more than 75% is considered strongly suggestive of cross-reactivity for vespid venoms [61,69,81,82,83].

IgE-inhibition assays are more accessible to perform than BAT. They can help distinguish between genuine double sensitization and cross-reactivity, even though FEIA inhibition presents some pitfalls in terms of technical procedures and costs [3,71].

A line blot immunoassay with allergens coating membrane strips in thin parallel lines as line blots (Euroline™; EUROIMMUN AG, Lübeck, Germany) may be used as a component-resolved multiparameter assay, based on immunoblot technology, with defined proteins as single venom recombinant allergen components for ssIgE antibody detection along with natural wve-s (Table 6). This in vitro oligoplex method uses alkaline phosphatase enzyme-labelled anti-IgE monoclonal antibodies and nitroblue tetrazolium chloride/5-bromo-4-chloro-3-indolylphosphate for colorimetric detection, with subsequent image acquisition and evaluation. If anti-CCD IgE antibodies are detected in a serum reflected in a positive CCD marker band, the serum should be re-incubated in the assay with an anti-CCD absorbent [74,84,85].

Multiplex ssIgE immunoassays enable the detection of the profile of IgE sensitizations against a wide array of allergens (more than one hundred from various sources). An ELISA-like patient-friendly allergen nano-bead array (FABER^®^, ADL s.r.l., Latina, Italy) allows the detection of IgE antibodies specifically recognizing allergens coupled to chemically activated nanobeads and immobilized on a biochip using an optical scanner and a particular software. This multiplex assay contains only two honeybee molecular allergens, the CCD-bearing Api m 1 and Api m 4, besides bee and wasp whole-venom extracts; therefore, it is not suitable for an accurate Hymenoptera venom allergy diagnosis. The ELISA-based macroarray immunoassay using state-of-the-art nano-bead technology as a molecular allergy explorer (ALEX^®^, MacroArray Diagnostics, Vienna, Austria) is the latest launched in vitro multiplex tool for precision medicine in allergy diagnosis. It uses several important hymenopteran venom allergen extracts and individual molecular components from honeybee, common wasp and paper wasp venoms (Table 7) spotted in a cartridge chip onto a nitrocellulose membrane, and anti-human IgE labelled with alkaline phosphatase. It is the first multiplex allergy test allowing simultaneous measurement of serum total IgE and ssIgE against many allergen extracts and molecular allergens. Its protocol integrates a potent CCD inhibitor during serum incubation, thus increasing the specificity of the results. The quantification of this colorimetric enzyme assay is achieved with a dedicated image explorer and software [9,74,86,87,88].

## 4. Basophil Activation Test (BAT) for Allergen Components in IgE-Mediated HVA

BAT can improve the accuracy of HVA diagnosis and the choice of VIT, but it should be performed within two days after the patient’s blood collection. Moreover, it is expensive, complex and requires special technical skills because the assay is not automated. Therefore, it is still not widely used [3].

In general terms, the BAT is considered a functional ex vivo assay that differentiates sensitization and relevant allergies. It usually uses CCR3 as a basophil identification marker and CD63 as a basophil activation marker. CD63 is not expressed on resting basophils and represents an accurate marker of anaphylactic degranulation. CD203c, constitutively expressed in low levels on resting basophils and representative of piecemeal degranulation, may also be used for BAT in patients with HVA as an activation marker with a slightly higher sensitivity but lower specificity than CD63. The sensitivity and specificity of the BAT with natural wve-s vary between around 85% and 100%, and there is no correlation between basophil activation and the clinical severity of the previously reported sting reaction. Performed not only with insect wve but also with individual allergen components, the BAT can help clinicians precisely detect clinically relevant insect venom and a proper decision regarding VIT [89,90,91,92,93].

The BAT is an ex vivo provocation assay based on allergen-induced activation of basophils that allows for the in vitro quantification of these activated cells by flow cytometry. The most widely used BAT assay is the FlowCAST^®^, a cellular allergy stimulation test (Bühlmann Laboratories AG, Schönenbuch, Switzerland) in which the in vitro basophil activation by Hymenoptera venom extracts or single venom components is assessed using a flow cytometry system. This established BAT technique utilizes monoclonal anti-FcεRI antibody and N-formyl-methionyl-leucyl-phenylalanine (fMLP) as IgE-dependent and IgE-independent positive controls, respectively, stimulation buffer containing IL-3 as a negative control, and conventional solutions with monoclonal antibodies conjugated with fluorochromes as staining reagents, anti-CCR3-PE/phycoerythrin and anti-CD63-FITC/fluorescein isothiocyanate. BAT results are usually reported as percentages of activated basophils (% CD63+ cells), sometimes also as mean fluorescent intensity (MFI). Furthermore, other BAT outcomes, such as the half-maximal allergen concentration as basophil sensitivity (EC50, CD-*sens*), the ratio of allergen-induced CD63 activation in comparison to an IgE-dependent positive control (CD63 ratio), and the area under the dose-response curve (AUC), may have additional clinical and therapeutic values [4,90,91,92,94].

The BAT with individual venom allergen components along with wve-s can confirm the diagnosis of HVA, especially in double-sensitized patients assessed by positive skin tests and ssIgE antibodies, and in patients with negative results with such routine allergy diagnostic tests. Up to 60% of the patients with HVA have ssIgE to both bee and wasp natural wve-s. Detection of ssIgE against honeybee venom rApi m 1 and wasp venom rVes v 5 reduces the genuine double sensitization to 50% of cases of double positivity, but the BAT with these CCD-free molecular allergens as stimuli may be critical in determining the culprit allergen source in some patients. The BAT consistently demonstrates lower levels of double positivity than other diagnostic methods [94,95,96].

Due to cross-reactivity, many patients with vespid venom allergy present double sensitization to *Vespula* and *Polistes* spp. wve-s, as revealed by skin tests and IgE immunoassay results. The BAT presents a higher sensitivity in such subjects than reciprocal IgE-inhibition assays, such as FEIA inhibition. In addition, the BAT has a good agreement with FEIA inhibition and can identify 100% of offender insects in cases with otherwise inconclusive results [81,82].

Moreover, a small proportion of patients (4–6%) with a clinical history of HVA report undetectable ssIgE and negative skin tests, considering the unethical nature of sting provocation tests under these circumstances. BAT has proven effective in diagnosing approximately 80% of these patients. Submaximal concentrations of recombinant allergens in the course of VIT have not been reported to assess the efficacy and tolerance of VIT [94,95,96,97,98].

The BAT is usually performed with Hymenoptera wve-s for FlowCAST^®^, and there is a trend to use in addition recombinant venom allergens (Table 8) which are much more specific when tested in the BAT compared to the respective ssIgE detection. The ability of functional specific IgE to mediate cellular responses might differentiate the allergic status from atopic sensitization. Moreover, the binding of different specific IgE to distinct epitopes of the allergens might be less facilitated in solid-phase IgE immunoassays compared to the BAT, various commercial Hymenoptera wve-s may differentially express different natural allergen components, including major ones, and extracts from diverse *Vespula* spp. may be mixed and thus may induce very different BAT activations, depending on the manufacturer and specific batches used. Therefore, recombinant venom allergens are needed to harmonize the BAT performed in different laboratories [94,95,99,100,101].

In HVA, the BAT is beneficial for diagnosing cases with an unclear history and complex IgE sensitization profiles, especially for vespid venoms. The BAT performed with rVes v 5, rVes v 3, and rVes v 1 appears to represent the best BAT approach in vespid venom-allergic subjects. rVes v 5 and rVes v 3 appear to increase sensitivity and specificity in the BAT compared with wasp wve-s in wasp-allergic patients. In contrast, in AmV-allergic patients, nApi m 1, rApi m 5, and rApi m 10 induce higher basophil activation than bee venom extracts only in single patients [4,94,95,100,101].

The AmV allergen rApi m 2 causes a moderate activation in Api m 2-sensitized AmV-allergic patients. However, neither hyaluronidase Pol d 2 nor Ves v 2.0201 reveals significant basophil activation in any Hymenoptera venom-allergic patient [94,102].

In scientific research, BAT is mainly used to characterize the allergenic components of Hymenoptera venoms and add important information regarding allergenicity and cross-reactivity. In the BAT, vespid-allergic patients reveal different activation profiles in response to the different venom Ag5 proteins: *Vespula vulgaris* rVes v 5, *Vespa crabro* rVesp c 5, *Polistes dominula* rPol d 5, *Polistes annularis* rPol a 5, *Dolichovespula maculata* Dol m 5, *Polybia scutellaris* rPoly s 5, and *Solenopsis invicta* Sol i 3. One-third of patients exhibit basophil activation only in response to rVes v 5 and/or rVesp c 5, and more than a half of them are activated by either all or different combinations of Ag5 molecules, demonstrating pronounced cross-reactivity of vespid venoms on a molecular level. Another venom allergen of *Polistes*, rPol d 3 reveals basophil activation in *Polistes* venom and/or *Vespula* venom-allergic patients from Southern Europe and honeybee and Vespula venom-allergic patients from Central Europe. The higher degree of cross-reactivity between Pol d 3 and Ves v 3 than between Pol d 3 and Api m 5 most likely reflects the sequence identity and conserved IgE epitopes [4,94,103].

Several BAT limitations should be mentioned. Up to about 10–15% of the patients are non-responders, with their basophils revealing no CD63 or CD203c activation to IgE-mediated allergen stimulation [96] or positive controls through anti-IgE and/or -FcεRI. This lack of response is believed to be due to differences in the intracellular signaling pathways, particularly in the expression of Syk. The BAT results in these cases are not interpretable. Moreover, the BAT can be helpful in patients with systemic mastocytosis and a history of anaphylaxis to Hymenoptera venoms, but with negative venom-specific IgE and skin tests. However, in cases where CCR3 is used as a basophil marker, basophils with low amounts of IgE on their surface are likely to be selected, explaining negative results with the BAT despite a clear history of HVA. Instead, positive results may be obtained using CD123/HLA-DR, CD45, and IgE [96,98,104].

Generally, the BAT is based on detecting allergen-induced basophil degranulation phenotype changes. As mentioned, different protocols have been developed using CCR3, CD123, CRTH2, CD203c, or anti-IgE to identify basophils. Such biomarkers are basically expressed on the basophil membrane, but not always specifically, and secondary markers are needed to exclude CRTH2+ T cells or CD123+ plasmacytoid dendritic cells. Among them, CCR3 and CD203c are exclusively expressed on resting basophils, and CD203c is up-regulated under cell activation. Degranulation is detected by surface expression of CD63 that is otherwise only expressed on the inner side of the granule membrane of resting basophils. The external expression of CD63 is correlated with the histamine release [93]. While the BAT assay represented by FlowCAST^®^ (Bühlmann Laboratories AG, Schönenbuch, Switzerland) is undoubtedly the most widely used, it is not the only one, and it is not restricted to being assessed using a specific flow cytometry system [91,92,93]. 

It is important to discuss how to standardize fluorescence detection between systems using CCR3/CD63 such as the FlowCast^®^ kit (Buhlmann, Schönenbuch, Switzerland) and CD203c/CD63 such as the BasoflowEx^®^ kit (Exbio, Praha, Czech Republic), and between clinical flow cytometry instruments, such as FACSCanto™ (Becton-Dickinson Biosciences), Cytomics™ FC 500, and Navios™ (Beckman Coulter), to better compare positive thresholds between clinical studies [93].

Furthermore, a multiplex BAT allows a reduction in consumables and reagents, equipment operation, and fewer samples for acquisition. Fluorescent labeling of major honeybee allergens, Api m 1 and Api m 2 with quantum dots, was reported in developing such a multiplex test. Quantum dot (Qdot) nanocrystals have broader excitation and narrower and brighter emission wavelengths than traditional fluorophores/dyes [105,106].

Besides the BAT, other cellular in vitro tests, which are not commonly used nowadays and less reliable than the BAT in indirectly assessing IgE-mediated HVA, are the LTC4 sulfidoleukotriene and histamine release tests measuring the two mediators in the cellular supernatant by ELISA and radioimmunoassay (RIA), respectively. These methods are based on the cross-linking of IgE on basophils, causing the release of both histamine and cysteinyl leukotrienes, especially after pretreatment with cytokines such as IL-3, but are unlikely to be available in the future due to European requirements for standardization of in vitro diagnosis [107,108,109].

## 5. Updated Algorithms for the Diagnosis of HVA Using Molecular Biomarkers in Clinical Practice

Component-resolved diagnostics (CRD) with recombinant or synthetic venom allergens and CCD markers, as a strategy of precision allergy molecular diagnostic application (PAMD@), is nowadays recommended for HVA in cases with multiple positive results from in vivo and/or in vitro allergy tests performed with different wve-s to discriminate between genuine sensitization and cross-reactivity, thus allowing clinicians to correctly identify the risks, to optimize venom selection for VIT and therefore to avoid treatment with double VIT. In the case of double-positive ssIgE detection to bee and vespid wve-s, there are different explanations, as mentioned before, such as clinical double sensitization to both venoms, irrelevant positive results due to specific IgE against CCDs or cross-reactivity of ssIgE to homologous allergens from both venoms, such as dipeptidyl peptidases, vitellogenins or hyaluronidases. Therefore, the patient’s serum screening for detecting ssIgE to CCDs is usually performed using CCD-rich substrates [3,41,107,110]. 

CRD is also recommended in patients with an inconclusive history for HVA, in cases of inconsistencies between the clinical history and classical diagnostic results, for identifying patients with hymenopteran venom-induced anaphylaxis who have negative test results to wve-s, including cases with negative test results with different wve-s despite a convincing clinical history and in mastocytosis patients [41,110].

**Figure 1:**
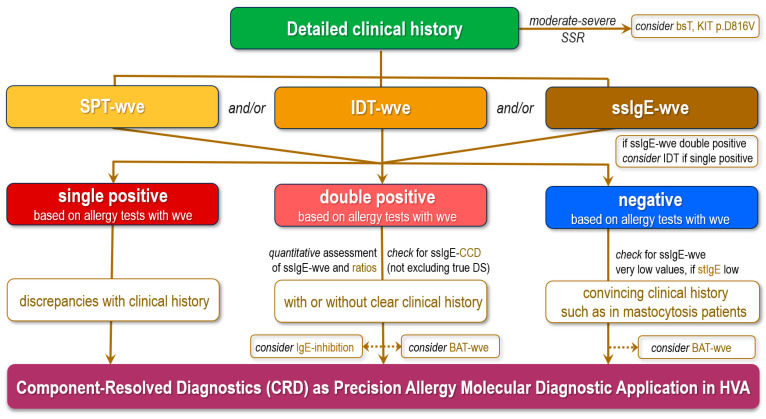
Algorithm for recommendations to perform IgE immunoassays using biomarkers for the molecular diagnosis of IgE-mediated HVA in clinical practice (adapted after Blank S et al., 2024; Hilger C et al., 2024 [9,41]). Note: HVA, Hymenoptera venom allergy; CRD, component-resolved diagnostics; SSRs, systemic sting reactions; bsT, basal serum tryptase; wve(-s), whole-venom extract(s); SPT, skin prick testing; IDT, intradermal testing; tsIgE, total serum IgE; ssIgE, serum specific IgE; CCDs, cross-reactive carbohydrate determinants; BAT, basophil activation test; FEIA, fluorescence enzyme immunoassay. This algorithm is a simplified interpretation with limitations. It cannot account for all individual parameters, circumstances, and potential decision pathways that should be considered when making HVA diagnosis.

An algorithm for recommendations to perform such IgE immunoassays in clinical practice is presented in Figure 1. The clinical diagnosis of HVA starts with a detailed patient history focusing on local or SSR evaluation, risk assessment, geographic location, and setting where the sting occurred. Attempts should be made to identify the culprit Hymenoptera species based on its appearance and behavior, sting embedment and avulsion, or sting autotomy. Because the majority of the general population cannot reliably distinguish between bees and vespids and many languages do not have separate names for the various wasps, patients may be shown pictures of the suspected stinging insects and, if necessary, biting dipterans to check whether the alleged insect can be identified. In the case of moderate to severe SSR, determining basal serum tryptase (bsT) by FEIA and KIT p.D816V(Asp816Val) mutation in peripheral blood using a highly sensitive allele-specific quantitative polymerase chain reaction (PCR) test is considered. About 5% of adult patients presenting for HVA evaluation are diagnosed with indolent systemic mastocytosis. The initial step in the allergy diagnosis of HVA, as previously mentioned, is represented by SPT-wve, IDT-wve, and/or determination of ssIgE to wve. There are no individual Hymenoptera venom components for in vivo skin testing approved by regulatory agencies. CRD may be performed using singleplex, multiparameter (oligoplex), or multiplex IgE immunoassays, and limitations should be considered because not all relevant allergens are available. CRD is recommended in different clinical scenarios, especially when there is double sensitization or multiple positive test results for different insect venoms, to differentiate between genuine sensitization(s) and clinically irrelevant cross-sensitization. If ssIgE-wve are double-positive for honeybee and wasp venoms, the result of IDT-wve if single positive is considered, as it is not influenced by CCDs. Determination of ssIgE to CCDs is important but has limited value compared with the cases of multiple sensitizations to pollen and foods of plant origin. In case of double positivity, reciprocal IgE-inhibition assays with wve-s or the BAT with wwe-s or molecular allergens can be performed besides molecular allergy IgE immunoassays. Other recommendations for CRD are in cases where negative results are achieved by allergy in vivo tests and/or ssIgE-wve of various insect venoms, despite a convincing clinical history, due to the potentially better sensitivity of CRD, such as in patients with mastocytosis. If the total serum IgE (tsIgE) is low (<30 kU/L), very low ssIgE levels between 0.10 kUA/L and 0.35 kUA/L can be regarded as positive. The BAT can be helpful in these cases. Furthermore, CRD is also useful in patients with an unclear or inconclusive history of allergic reaction(s) and when there are discrepancies regarding the culprit insect(s) between the clinical history and the results of standard diagnostic using wves. Finally, it should be stated that the mentioned diagnosis tests may be combined to increase sensitivity [3,9,41,107].

**Figure 2:**
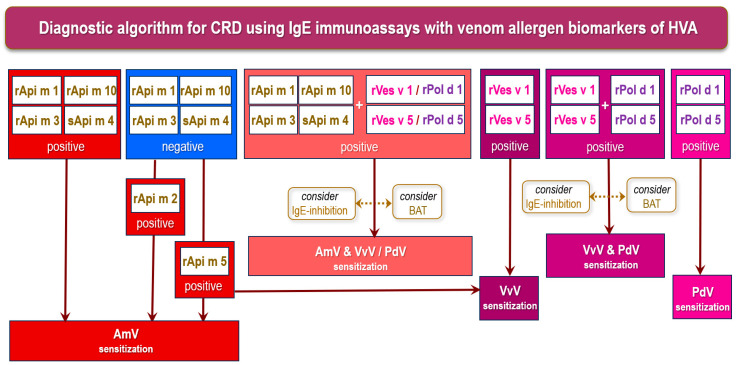
Diagnostic algorithm for CRD with venom allergen biomarkers of IgE-mediated HVA in clinical practice (adapted after Blank S et al., 2024; Hilger C et al., 2024 [9,41]). Note: HVA, Hymenoptera venom allergy; BAT, basophil activation test; CRD, component-resolved diagnostics; honeybee or *Apis mellifera* venom (AmV); European common wasp or yellow jacket *Vespula vulgaris* venom (VvV); European paper wasp *Polistes dominula* venom (PdV). In CRD, not all allergens in a group must test positive to indicate sensitization, with individual reactivities being sufficient to demonstrate sensitization. Despite the potential of CRD, a detailed clinical history, skin testing with wve-s, and ssIgE to wve-s form a central basis for HVA diagnosis. Additionally, IgE-inhibition assays and BAT can be helpful in unraveling primary sensitizations. This algorithm is a simplified interpretation with limitations. It cannot account for all individual parameters, circumstances, and potential decision pathways that should be considered when making a final diagnosis of HVA.

An updated diagnostic algorithm for CRD with venom allergen biomarkers of IgE-mediated HVA in clinical practice is presented in Figure 2. This algorithm may be used for a more accurate diagnostic of honeybee or *Apis mellifera* venom (AmV) allergy, European common wasp or yellow jacket *Vespula vulgaris* venom (VvV) allergy, and European paper wasp *Polistes dominula* venom (PdV) allergy, but it represents a simplified interpretation with many boundaries. It cannot account for all individual parameters, situations, and potential decision pathways that should be considered when making a concluding diagnosis of HVA. The AmV allergens, Api m 1, Api m 3, Api m 4, and Api m 10, are considered pivotal as allergen biomarkers for detecting primary sensitization to AmV. Api m 2 cross-reactivity with vespid venom hyaluronidases is considered limited outside CCD moieties. Thus, rApi m 2 may contribute to detecting genuine AmV sensitization. Mono-sensitization to Api m 5 may indicate cross-reactivity to vespid venoms, but in cases where AmV allergy is highly likely, and other tests are negative, the use of Api m 5 still remains a diagnostic option to be considered. When used alongside vespid phospholipases A1 (Ves v 1/Pol d 1) and antigens 5 (Ves v 5/Pol d 5), these biomarkers facilitate accurate differentiation between allergies to AmV and VvV. The comparison of the levels of ssIgE with the homologous allergen pairs, Ves v 5 and Pol d 5, and Ves v 1 and Pol d 1, enables a reliable identification of the allergy-eliciting venom in many of double-sensitized patients. However, a definite resolution of cross-reactivity and primary sensitization is hardly possible using only available vespid allergens to diagnose VvV and PdV allergy. Moreover, FEIA inhibition assays and the BAT represent helpful diagnostic tools for a more accurate assessment of primary sensitization [4,9,41,75,111].

Although double sensitization to AmV and VvV is frequent, genuine double allergy is uncommon. Current recommendations for assessing double-positive serum diagnostic tests in venom-allergic subjects do not adequately consider quantitative ssIgE levels. IgE sensitization to venom-specific biomarker allergens is frequent in nonallergic subjects, it is not a proof of genuine allergy and should not be equated with an indication for double VIT in double-sensitized patients. In individuals with anaphylaxis to one venom and asymptomatic IgE sensitization to another, higher ssIgE to wve may indicate the culprit insect. A 5:1-dominant ssIgE level is a robust indicator of the culprit venom (AmV or VvV) within the first 5 years after the index sting. Therefore, additional testing with CRD is particularly recommended to identify the culprit insect when no ssIgE is detected to wve and in the case of double sensitization to AmV and VvV at a ratio of less than 5:1 [112].

## 6. Discussions on Challenges and Clinical Implications in HVA Patient Management

Integration of presented algorithms in diagnosing HVA using molecular biomarkers in clinical practice currently needs some comments and discussions about limitations. CRD can be expensive, thus limiting molecular diagnosis for some patients or healthcare facilities. Not all identified molecular allergens, either native purified components or recombinant CCD-free molecules, are available in commercial IgE immunoassays. Most relevant molecular allergens, rApi m 1, rApi m 2, rApi m 3, rApi m 5, and rApi m 10, are considered as AmV major allergens, although with significant prevalence variations according to the patient population, geographical regions, and method of detection.

Almost three-quarters of patients are sensitized to multiple allergens within the AmV. IgE immunoassays using these recombinant molecular allergens allow about 95% diagnostic sensitivity. These particular allergen molecules can serve as biomarkers for a primary, genuine, family- or species-specific IgE-mediated sensitization and provide improved analytical specificity compared with allergen wve-s testing. Although the diagnostic specificity of IgE to rApi m 1 for HBV allergy has been constantly reported at very high levels of 97–100%, its diagnostic sensitivity may be as low as 57%, and a panel with selected AmV relevant allergens, Api m 1, 2, 3, 5, and 10, is also low at 72%. Therefore, even if specific IgE antibodies to all these molecules are undetected, AmV allergy cannot be excluded. Moreover, honeybee and bumble bee venoms are highly cross-reactive, and differential diagnosis is not currently possible using CRD. Regarding wasp venom, the detection of Ves v 5-specific IgE is a hallmark of genuine sensitization to Vespid venom, exhibiting a diagnostic specificity of 92–100%, while their diagnostic sensitivity ranges from 82% to 98% in VvV allergic patients. If both, Ves v 1 and 5 are negative, vespid venom allergy is unlikely. Nevertheless, some relevant cross-reactive bee and vespid allergens, such as vitellogenins Api m 12 and Ves v 6, as well as dipeptidyl peptidase Ves v 3, are still unavailable for routine diagnosis. The ssIgE antibodies against major allergens, rApi m 1, rApi m 3, rApi m 4 or rApi m 10, indicate primary AmV sensitization, while ssIgE tor Api m 2 may be an additional helpful biomarker to detect primary AmV sensitization, with the need to interpret results with care in the context of clinical history. The predominance of IgE sensitization to Api m 1 and Api m 10 reinforces the need to always include these molecular allergens in diagnostic panels. The identification and confirmation of additional allergens, such as protease inhibitor Api m 6, and the stability of icarapin Api m 10 in therapeutic preparations further inform management strategies for HBV allergy. In addition, ssIgE to cross-reactive rApi m 5 does not exclude primary vespid venom allergy. By contrast, ssIgE to rVes v 1/rPol d 1, and rVes v 5/rPol d 5 indicate primary vespid venom sensitization, while these are not reliable markers to accurately differentiate between primary sensitization to common wasp/yellow jacket and/or European paper wasp venom. The homologous allergen pairs of *Vespula* and *Polistes* spp., Ves v 5 and Pol d 5, and Ves v 1 and Pol d 1, enable reliable identification of the allergy-eliciting venom in 67% of double-sensitized patients. However, Ves v 5 exhibits extensive cross-reactivity with other Ag5 proteins from *Vespa crabro* and *Dolichovespula* spp. venoms; therefore, it cannot be used to discriminate between such vespid sensitization or cross-reactivity. Concerning Solenopsis spp. venoms, although unique peptides, Sol i 2 and Sol r 2, may be used to identify such invasive insects entomologically with a lateral flow immunoassay, currently, there are not commercially available IgE immunoassays using such imported fire ant molecular biomarkers. Finally, the updated presented algorithms are simplified interpretations that cannot account for all individual parameters, circumstances, and potential decision pathways that should be considered when making a final diagnosis of HVA [4,9,41,113,114].

In general, molecular diagnosis of HVA presents significant challenges, particularly in cases of complex sensitization patterns. Sensitivity and specificity issues are prominent concerns. Standard allergy tests with wve-s sometimes reveal IgE sensitization to more than one Hymenoptera venom, posing difficulties in identifying the primary sensitizer. More than half of patients with HVA have positive results for both AmV and VvV/PdV in serological tests. Interpreting complex IgE sensitization profiles is further complicated by cross-reactivity between venom components. Different tests may yield varying results, sometimes providing conflicting information. This variability emphasizes the need for multiple diagnostic approaches to achieve accurate diagnosis [38,82,83,115,116].

HVA is an excellent illustration in which ssIgE to individual allergens serve as biomarkers for genuine sensitization to AmV (Api m 1, Api m 3, Api m 4, Api m 10) or VvV (Ves v 1, Ves v 5), while IgE to homologous allergens (such as the hyaluronidases Api m 2 and Ves v 2; the dipeptidyl peptidase IV Api m 5 and Ves v 3 and the vitellogenins Api m 12 and Ves v 6) indicate positive wve-based test results based on cross-reactivity [4].

The presence of ssIgE against CCDs hinders the interpretation of the results of in vitro assays using hymenopteran wve-s and native allergen glycoproteins which carry CCDs, namely α1,3-linked fucose at the innermost N-acetylglucosamine of the core structure of N-linked glycans, which are not found on human proteins. Therefore, a significant proportion of venom-allergic patients, generally more than 20%, develop ssIgE against these CCDs in addition to the allergen protein-specific ones. IgE antibodies against CCDs have no clinical relevance established so far. Therefore, using recombinant CCD-free components and potent CCD inhibitors is beneficial for IgE immunoassays. Due to the recombinant production in *E. coli*, the Hymenoptera venom single allergens do not exhibit CCD [4,24,90]. Moreover, the BAT with wve-s and/or components can help differentiate between genuine double sensitization and cross-reactivity. The discrepancies between ssIgE and BAT results vary depending on the allergen tested [24,90,117,118,119,120].

When interpreting results, it is also imperative to consider phylogenetic relationships and cross-reactivities due to structural homologies between allergen components from different species. Therefore, a molecular diagnosis approach can lead to more precise identification of clinically relevant venom and guide appropriate VIT decisions [3,70,117,118,120]. Hyaluronidases, ubiquitous components of various species’ venoms, span across taxonomic orders, including Hymenoptera (bees, wasps, hornets, and ants), but also snakes, spiders, and even the platypus. As previously mentioned, Api m 2 is a key allergen in AmV allergy, capable of activating basophils in susceptible patients and serving as a critical biomarker for primary AmV sensitization. While vespid homologs Pol d 2, Ves v 2.0101, and Ves v 2.0201 appear to play a minor role as allergens, their relevance cannot be completely ruled out due to occasional cases of primary sensitization. It is important to underline the limited cross-reactivity of Api m 2 with such vespid homologs in the absence of CCDs [119].

Geographical variations and rare insect species may play a significant role in diagnosing and treating HVA, adding complexity to an already challenging field, along with the imprecise terminology for wasps in many languages. While yellow jackets are prominent in the Northern Hemisphere, paper wasps are more significant in the United States and Mediterranean regions. Moreover, other wasp species, such as *Polybia paulista*, are significant in South America (Southwest Brazil, Paraguay, and North Argentina). Additionally, allergies to hornet stings are common and potentially increasing due to the spread of invasive species, as in the case of *Vespa velutina nigrithorax*, commonly known as the Asian hornet, originally endemic to Asia, which has spread across several European countries since its introduction to France in 2004, including Spain, Portugal, Italy, and others [9,38,118]. Its major venom allergens reveal high structural homology with those of *Vespa crabro* and *Vespula* spp., but less similarity with *Polistes dominula*, with impact on diagnosis and treatment [38,70]. Due to similarities between venoms of hornet *Vespa crabro* and wasp *Vespula* spp., patients with hornet venom anaphylaxis are often treated similarly to wasp-allergic patients [121].

Clinical reports on patients stung by rare or exotic Hymenoptera emphasize the significance of the HVA diagnosis for both tourists/travelers and residents in areas with invasive species. SSRs due to rare species of the Apidae family are scarce. However, *Apis dorsata* is the largest and most aggressive honey bee in Sri Lanka, where a fatal case after a carpenter bee *Xylocopa tranquebarica* sting has also been reported. Stings of rare species of the Vespidae family are uncommon. Worldwide distributed Scoliid solitary wasps rarely sting humans under natural conditions. Anaphylaxis after *Scolia flavifrons* (also known as *Megascolia maculata* subsp. *flavifrons*) was reported in Italy. Regarding locally important wasps, besides *Polybia paulista* from South America and *Vespa velutina*, which is endemic to Asia and invasive in South Europe, *Vespa affinis*, a common hornet in tropical and subtropical Asia, and *Vespa orientalis*, found in Southwest Asia and Northeast Africa, rarely induce anaphylaxis. Furthermore, the paper wasp *Ropalidia marginata*, extending from Pakistan, India, and Sri Lanka to Queensland, New Guinea and some eastern Pacific islands, has been linked to anaphylactic reactions [122].

The potential underrepresentation of specific molecules in conventional wve-s, such as icarapin in AmV, further emphasizes the importance of considering various venom allergen components. Venom characteristics, geographical differences, and invasive species highlight the need for region-specific diagnostic approaches [49,101]. Moreover, hymenopteran venoms are considered the most common triggers of work-related anaphylaxis. Occupational groups at risk for HVA include beekeepers, forestry workers, farmers, gardeners, landscapers, workers in greenhouses, firefighters, bakery shop assistants, pest control and construction workers, drivers operating open vehicles and outdoor workers active in areas with high insect activity [123]. 

Finally, we have to mention that although the Hymenoptera insects comprise more than 100,000 known species worldwide, only a subgroup of female insects from the Aculeata infraorder can inject venom when stinging with their modified ovipositor stinger. To assess IgE sensitization to Hymenoptera venoms, allergists usually determine concentrations of ssIgE against AmV, VvV, PdV and their molecular components. If negative results are obtained shortly (less than 2 weeks) after the sting reaction, the diagnosis tests shall be repeated (no sooner than 4-6 weeks after the sting reaction). In case of a suspected sting reaction caused by other Hymenoptera, the IgE immunoassay assessment shall also be directed against the corresponding other venom. Notably, changes in climatic conditions in Europe could lead to the emergence or spread of previously non-native insect species. Moreover, specific IgG antibodies to Hymenoptera venom may be pathophysiologically relevant in patients with serum sickness-type or other unusual sting reactions, but their determination should not be used to assess the need for treatment of HVA. A high concentration of specific IgG antibodies is an epiphenomenon of allergen exposure, including immunotherapy, but does not prove protection against future systemic sting reactions [107].

While CRD has significantly improved the diagnosis of HVA, there remains a need for better assessment methods and additional molecular biomarkers. These improvements could enhance therapy effectiveness, help identify potential non-responders to VIT, predict patients at risk of severe side effects and evaluate immunological tolerance after VIT discontinuation. Ultimately, these advancements will enable more personalized treatment strategies and the selection of the most appropriate venom preparation for each patient [1,124,125].

## 7. Conclusions

CRD with molecular venom allergens is an indispensable strategy for precision allergy diagnostic application. It is nowadays recommended in HVA, especially in cases with multiple positive results from in vivo and in vitro allergy tests performed with different natural wve-s to discriminate between genuine sensitization and cross-sensitizations. Thus, it allows clinicians to correctly identify risks and optimize venom selection for allergen immunotherapy. Knowledge of molecular allergen biomarkers and updated algorithms are essential in allergy clinical practice. Additional biomarkers are needed to monitor therapy effectiveness better, identify non-responders to VIT, predict patients at risk for severe side effects and assess immunological tolerance after VIT discontinuation.

## Figures and Tables

**Table 1:** Molecular hymenopteran venom allergens from eusocial bees belonging to the Apidae family, listed in the WHO/IUIS database (adapted from NCBI Taxonomy and Dramburg et al., 2023 [4]).

Hymenoptera Insect from the Apidae Family	Allergen	Biochemical Name	MW (kDa)
Tribe Apini (honeybees)			
*Apis mellifera* (European honeybee, Western honeybee)	Api m 1 ^#^	Phospholipase A2	16
	Api m 2	Hyaluronidase	44
	Api m 3	Acid phosphatase	43
	Api m 4	Melittin	3
	Api m 5	Dipeptidyl peptidase IV	100
	Api m 6	Protease inhibitor	8
	Api m 7	CUB serine protease ^##^	39
	Api m 8	Carboxylesterase	70
	Api m 9	Serine carboxypeptidase	60
	Api m 10	Icarapin variant 2	50–55
	Api m 11	Major royal jelly protein	50 *
	Api m 12	Vitellogenin	200
*Apis cerana* (Asian honeybee)	Api c 1	Phospholipase A2	16
*Apis dorsata* (Southeast Asian giant honeybee)	Api d 1	Phospholipase A2	16
Tribe Bombini (bumble bees)			
*Bombus terrestris* (European bumblebee)	Bom t 1	Phospholipase A2	16
	Bom t 4	Protease	27
*Bombus pensylvanicus* (American bumblebee)	Bom p 1	Phospholipase A2	16
	Bom p 4	Protease	27

Note: MW (kDa), molecular weight in kDa; * deglycosylated form; ^#^ Unique isoallergen, Api m 1.0101, has been recently included in the World Health Organization (WHO) and International Union of Immunological Societies (IUIS) Allergen database; ^##^ CUB domain (for complement C1r/C1s, Uegf, Bmp1) is a structural protein motif.

**Table 2:** Molecular hymenopteran venom allergens from eusocial wasps belonging to the Vespidae family, from the Vespinae subfamily, listed in the WHO/IUIS Allergen database (adapted from NCBI Taxonomy and Dramburg et al., 2023 [4]).

Hymenoptera Insect from the Vespidae Family, the Vespinae Subfamily	Allergen	Biochemical Name	MW (kDa)
Genus *Vespinae* (short-headed wasps, yellow jackets, with nests usually underground/cavities)			
*Vespula vulgaris* (European common wasp, European common yellow jacket)	Ves v 1 ^#^	Phospholipase A1B	34
	Ves v 2	Hyaluronidase	38
	Ves v 3	Dipeptidyl peptidase IV	100
	Ves v 5 ^#^	Wasp venom antigen 5	23
	Ves v 6	Vitellogenin	200
*Vespula germanica* (German wasp, German yellow jacket)	Ves g 5	Wasp venom antigen 5	23
*Vespula maculifrons* (Eastern North American yellow jacket)	Ves m 1	Phospholipase A1B	34
	Ves m 2	Hyaluronidase	46
	Ves m 5	Wasp venom antigen 5	23
*Vespula pensylvanica* (Western North American yellow jacket)	Ves p 5	Wasp venom antigen 5	23
*Vespula squamosa* (Southern North American yellow jacket)	Ves s 1	Phospholipase A1B	34
	Ves s 5	Wasp venom antigen 5	23
*Vespula flavopisola* (North American downy yellow jacket)	Ves f 5	Wasp venom antigen 5	23
*Vespula vidua* (North American widow yellow jacket)	Ves vi 5	Wasp venom antigen 5	23
Genus *Dolichovespula* (long-headed wasps, hornet-like yellow jackets, with nests usually aerial)			
*Dolichovespula arenaria* (North American common yellow hornet, common aerial yellowjacket)	Dol a 5	Wasp venom antigen 5	23
*Dolichovespula maculata* (North American bald-faced hornet, white-faced hornet, blackjacket, white-tailed hornet, bald-faced aerial yellowjacket, bull wasp)	Dol m 1	Phospholipase A1B	34
	Dol m 2	Hyaluronidase	42
	Dol m 5	Wasp venom antigen 5	23
Genus *Vespa* (hornets, with nests aerial/underground/cavities)			
*Vespa crabro* (European hornet)	Vesp c 1	Phospholipase A1B	34
	Vesp c 5	Wasp venom antigen 5	23
*Vespa velutina* (Asian yellow-legged hornet, Asian predatory wasp invasive in Europe)	Vesp v 1	Phospholipase A1	36.1
	Vesp v 5	Wasp venom antigen 5	23
*Vespa magnifica* (Asian giant hornet)	Vesp ma 2	Hyaluronidase	35
	Vesp ma 5	Wasp venom antigen 5	25
*Vespa mandarinia* (Asian giant hornet)	Vesp m 1	Phospholipase A1B	34
	Vesp m 5	Wasp venom antigen 5	23

Note: MW (kDa), molecular weight in kDa; *Vespula alascensis* (North American common yellow jacket) was recently recognized as a distinct species from *Vespula vulgaris*; Eurasian *Dolichovespula* wasps include tree wasp *Dolichovespula sylvestris*, median wasp *Dolichovespula media*, and Saxon wasp *Dolichovespula saxonica*. ^#^ Unique isoallergens, Ves v 1.0101 and Ves v 5.0101, have been recently included in the WHO and IUIS Allergen database.

**Table 3:** Molecular hymenopteran venom allergens from primarily eusocial wasps belonging to the Vespidae family, from the Polistinae subfamily, listed in the WHO/IUIS Allergen database (adapted from NCBI Taxonomy and Dramburg et al., 2023 [4]).

Hymenoptera Insect from the Vespidae Family, the Polistinae Subfamily	Allergen	Biochemical Name	MW (kDa)
Tribe Polistini (paper wasps)			
*Polistes dominula* (also known as *Polistes dominulus*) (European paper wasp, Mediterranean paper wasp)	Pol d 1 ^#^	Phospholipase A1	34
	Pol d 2	Hyaluronidase	50
	Pol d 3	Dipeptidyl peptidase IV	100
	Pol d 4	Serine protease	33
	Pol d 5 ^#^	Wasp venom antigen 5	23
*Polistes gallicus* (French paper wasp)	Pol g 1	Phospholipase A1	33.475
	Pol g 5	Wasp venom antigen 5	24
*Polistes fuscatus* (North American dark paper wasp)	Pol f 5	Wasp venom antigen 5	23
*Polistes exclamans* (North American Guinea paper wasp)	Pol e 1	Phospholipase A1	34
	Pol e 4	Serine protease	33
	Pol e 5	Wasp venom antigen 5	23
*Polistes annularis* (North American ringed paper wasp)	Pol a 1	Phospholipase A1B	34
	Pol a 2	Hyaluronidase	38
	Pol a 5	Wasp venom antigen 5	23
*Polistes metricus* (North American metric paper wasp)	Pol m 5	Wasp venom antigen 5	23
Tribe Epiponini (Neotropical wasps)			
*Polybia paulista* (South American swarm-founding wasp)	Poly p 1	Phospholipase A1	34
	Poly p 2	Hyaluronidase	33
	Poly p 5	Wasp venom antigen 5	21.19
*Polybia scutellaris* (South American wasp camoati)	Poly s 5	Wasp venom antigen 5	23

Note: MW (kDa), molecular weight in kDa. ^#^ Isoallergens, Pol d 1.0101, Pol d 1.0102, Pol d 1.0103, Pol d 1.0104, and Pol d 5.0101, have been recently included in the WHO and IUIS Allergen database.

**Table 4:** Molecular hymenopteran venom allergens from stinging ants (the Formicidae family), listed in the WHO/IUIS database (adapted from NCBI Taxonomy and Dramburg et al., 2023 [4]).

Hymenoptera Insect from the Formicidae Family	Allergen	Biochemical Name	MW (kDa)
Tribe Solenopsidini			
*Solenopsis invicta * (red imported fire ant in Southern US, native from South America)	Sol i 1	Phospholipase A1B	18
	Sol i 2	Ant venom, group 2	14
	Sol i 3	Venom antigen 5	26
	Sol i 4	Ant venom, group 4	12
*Solenopsis richteri * (black imported fire ant in Southeast US, native from South America)	Sol r 2	Ant venom, group 2	13
	Sol r 3	Venom antigen 5	24
*Solenopsis geminata* (Central and South American/tropical native fire ant)	Sol g 2	Ant venom, group 2	13
	Sol g 3	Venom antigen 5	24
	Sol g 4	Ant venom, group 4	12
*Solenopsis saevissima* (South American native fire ant)	Sol s 2	Ant venom, group 2	13
	Sol s 3	Venom antigen 5	24
Tribe Ponerini			
*Brachyponera*/*Pachycondyla chinensis* (Asian needle ant)	Pac c 3	Venom antigen 5	23
Tribe Myrmeciini			
*Myrmecia pilosula* (Australian jumper ant, jack jumper ant, hopper ant)	Myr p 1	[Ile5]pilosulin-1	7.5, 5.5
	Myr p 2	pilosulin-3	8.5, 2–4
	Myr p 3	pilosulin-4.1	8.2

Note: MW (kDa), molecular weight in kDa.

**Table 5:** Insect venom extracts and components used in reference singleplex sIgE immunoassays.

Venom Allergen	Latin Name, Protein Group	Code	Singleplex Assays	
Hymenoptera natural wve-s				
Honey bee venom	wve *Apis mellifera*	i1	ImmunoCAP^®^ FEIA	Immulite^®^ CLIA
Bumble bee venom	wve *Bombus terrestris*	i205	ImmunoCAP^®^ FEIA	
Common wasp/yellow jacket venom	wve *Vespula vulgaris*	i3	ImmunoCAP^®^ FEIA	Immulite^®^ CLIA
White-faced hornet venom	wve *Dolichovespula maculata*	i2	ImmunoCAP^®^ FEIA	Immulite^®^ CLIA
Yellow hornet venom	wve *Dolichovespula arenaria*	i5	ImmunoCAP^®^ FEIA	Immulite^®^ CLIA
European hornet venom	wve *Vespa crabro*	i75	ImmunoCAP^®^ FEIA	Immulite^®^ CLIA
Asian hornet venom	wve *Vespa velutina*	U1223 *	ImmunoCAP^®^ FEIA	
North American paper wasps	wve *Polistes* spp.	i4 **	ImmunoCAP^®^ FEIA	Immulite^®^ CLIA
European paper wasp venom	wve *Polistes dominula*	i77	ImmunoCAP^®^ FEIA	
Red imported fire ant venom	wve *Solenopsis invicta*	i70	ImmunoCAP^®^ FEIA	Immulite^®^ CLIA
Hymenoptera venom allergen components				
rApi m 1 honey bee venom	phospholipase A2 *Apis mellifera*	i208	ImmunoCAP^®^ FEIA	Immulite^®^ CLIA
rApi m 2 honey bee venom	hyaluronidase *Apis mellifera*	i214	ImmunoCAP^®^ FEIA	Immulite^®^ CLIA
rApi m 3 honey bee venom	acid phosphatase *Apis mellifera*	i215	ImmunoCAP^®^ FEIA	
sApi m 4 honey bee venom	melittin *Apis mellifera*	U1273 *	ImmunoCAP^®^ FEIA	
rApi m 5 honey bee venom	dipeptidyl peptidase *Apis mellifera*	i216	ImmunoCAP^®^ FEIA	
rApi m 10 honey bee venom	icarapin *Apis mellifera*	i217	ImmunoCAP^®^ FEIA	
rVes v 1 common wasp venom	phospholipase A1 *Vespula vulgaris*	i211	ImmunoCAP^®^ FEIA	
rVes v 5 common wasp venom	venom antigen 5 *Vespula vulgaris*	i209	ImmunoCAP^®^ FEIA	Immulite^®^ CLIA
rPol d 5 paper wasp venom	venom antigen 5 *Polistes dominula*	i210	ImmunoCAP^®^ FEIA	

Note: FEIA, fluorescence enzyme immunoassay, CLIA, chemiluminescence immunoassay; wve(-s), whole venom extract(s); r, CCD-free recombinant, s, synthetic; * R.U.O., Research Use Only; ** North American *Polistes* wasps (paper wasps): *P. annularis*, *P. exclamans*, *P. fuscatus*, and *P. metricus*; fire ants *Solenopsis* spp.: *S. richteri* and *S. invicta*; nMUXF3 sugar epitope from bromelain (o214) is the cross-reactive carbohydrate determinant or CCD-marker in both singleplex sIgE immunoassays.

**Table 6:** Insect venom extracts and allergen molecular components used in multiparameter line blot IgE immunoassays.

Venom Allergen	Latin Name, Protein Group	Code	Multiparameter Euroline™ Assays
Hymenoptera natural wve-s			
Honey bee venom	wve *Apis mellifera*	i1	DPA-Dx * insect venoms 3, SE1
Common wasp venom	wve *Vespula vulgaris*	i3	DPA-Dx * insect venoms 3, SE1
Hornet venom	wve *Vespa crabro*	i75	DPA-Dx * insect venoms 3, SE1
Polistes venom	wve *Vespa dominula*	i77	DPA-Dx * insect venoms SE1
Hymenoptera venom allergen components			
rApi m 1 honey bee venom	phospholipase A2 *Apis mellifera*	i208	DPA-Dx * insect venoms 3, SE1
rApi m 2 honey bee venom	hyaluronidase *Apis mellifera*	i213	DPA-Dx * insect venoms 3, SE1
rApi m 10 honey bee venom	icarapin variant 2 *Apis mellifera*	i216	DPA-Dx * insect venoms 3, SE1
rVes v 1 common wasp venom	phospholipase A1 *Vespula vulgaris*	i211	DPA-Dx * insect venoms 3, SE1
rVes v 5 common wasp venom	venom antigen 5 *Vespula vulgaris*	i209	DPA-Dx * insect venoms 3, SE1
rPol d 1 paper wasp venom	phospholipase A1 *Polistes dominula*	i220	DPA-Dx * insect venoms SE1
rPol d 5 paper wasp venom	venom antigen 5 *Polistes dominula*	i210	DPA-Dx * insect venoms SE1

Note: Euroline™ line blot immunoassay for insect venom allergy; wve(-s), whole venom extract(s); * defined partial allergens diagnostics (DPA-Dx) include an integrated unique CCD; panel of allergens: insect venoms 3 and insect venoms SE1 (Southern Europe 1); r, CCD-free recombinant.

**Table 7:** Hymenoptera insect venom extracts and allergen molecular components used in the latest multiplex macroarray IgE immunoassay.

Venom Allergen	Latin Name, Protein Group	Code	Multiplex Assay
Hymenoptera natural wve-s			
Honeybee venom	wve *Apis mellifera*	i1	ALEX2^®^
Common wasp venom	wve *Vespula vulgaris*	i3	ALEX2^®^
Long-headed wasp venom	wve *Dolichovespula* spp.	i25	ALEX2^®^
Paper wasp venom	wve *Polistes* spp.	i4	ALEX2^®^
Fire ant venom	wve *Solenopsis richteri* & *Solenopsis invicta*	i70	ALEX2^®^
Hymenoptera venom allergen components			
nApi m 1 honeybee venom	phospholipase A2 *Apis mellifera*	i208	ALEX2^®^
rApi m 10 honeybee venom	icarapin variant 2 *Apis mellifera*	i217	ALEX2^®^
rVes v 1 common wasp venom	phospholipase A1 *Vespula vulgaris*	i211	ALEX2^®^
rVes v 5 common wasp venom	venom antigen 5 *Vespula vulgaris*	i209	ALEX2^®^
rPol d 5 paper wasp venom	venom antigen 5 *Polistes dominulus*	i210	ALEX2^®^

Note: ALEX2^®^ is the improved successor product of ALEX^®^ = Allergy Xplorer ELISA-based macroarray immunoassay, its protocol integrates a CCD inhibitor; wve(-s), whole venom extract(s); n, natural purified, r, CCD-free recombinant; North American *Polistes* spp.: *P. annularis*, *P. exclamans*, *P. fuscatus*, and *P. metricus*; North American *Dolichovespula* spp.: *D. maculata* and *D. arenaria*; human lactoferrin rHom s LF (o214) is a CCD marker. Interestingly, the human lactoferrin produced in genetically engineered rice is glycosylated with plant CCD.

**Table 8:** Examples of insect venom allergen extracts and molecular components used in BAT.

Venom Allergen	Latin Name, Protein Group	Code	Source	BAT Assay
Hymenoptera natural wve-s				
Honey bee venom	wve *Apis mellifera*	BAG2-I1	native venom	FlowCAST^®^
Wasp venom	wve *Vespula* spp.	BAG2-I3	native venom	FlowCAST^®^
Hornet venom	wve *Vespa crabro*	BAG2-I75	native venom	FlowCAST^®^
Paper wasp venom	wve *Polistes dominula*	BAG2-I77	native venom	FlowCAST^®^
Hymenoptera venom allergen components				
nApi m 1 honey bee venom	phospholipase A2 *Apis mellifera*	i208	native venom	FlowCAST^®^
rApi m 10 honey bee venom	icarapin *Apis mellifera*	i217	Sf9 insect cells or *E. coli*	FlowCAST^®^
rVes v 1 common wasp venom	phospholipase A1 *Vespula vulgaris*	i211	Sf9 insect cells	FlowCAST^®^
rVes v 5 common wasp venom	venom antigen 5 *Vespula vulgaris*	i209	Sf9 insect cells	FlowCAST^®^

Note: FlowCAST^®^ is a cellular allergy stimulation test in which in vitro basophil activation by allergen is assessed using a flow cytometry system; wve(-s), whole venom extract(s); n, natural purified, r, recombinant; The Hymenoptera venom hyaluronidases Api m 2, Pol d 2, and Ves v 2.0201 may also be recombinantly produced in Sf9 (*Spodoptera frugiperda*) insect cells. The Sf9 cells add carbohydrate modifications to the recombinant protein allergens, however, the attached carbohydrate structure lacks the α-1,3-core-fucosylation, which is the molecular basis for CCD reactivity.

## References

[1] Blank S., Grosch J., Ollert M., Bilò M.B. (2020). Precision Medicine in Hymenoptera Venom Allergy: Diagnostics, Biomarkers, and Therapy of Different Endotypes and Phenotypes. Front. Immunol..

[2] Schneider S., Gasteiger C., Wecker H., Höbenreich J., Biedermann T., Brockow K., Zink A. (2023). Successful usage of a chatbot to standardize and automate history taking in Hymenoptera venom allergy. Allergy.

[3] Sturm G.J., Arzt-Gradwohl L. (2024). An algorithm for the diagnosis and treatment of Hymenoptera venom allergy, 2024 update. Allergy.

[4] Dramburg S., Hilger C., Santos A.F., Vecillas L.d.L., Aalberse R.C., Acevedo N., Aglas L., Altmann F., Arruda K.L., Asero R. (2023). EAACI Molecular Allergology User’s Guide 2.0. Pediatr. Allergy Immunol..

[5] Giovannini M., Mori F., Barni S., Saretta F., Arasi S., Castagnoli R., Liotti L., Mastrorilli C., Pecoraro L., Caminiti L. (2024). Hymenoptera venom allergy in children. Ital. J. Pediatr..

[6] Worm M., Moneret-Vautrin A., Scherer K., Lang R., Fernandez-Rivas M., Cardona V., Kowalski M.L., Jutel M., Poziomkowska-Gesicka I., Papadopoulos N.G. (2014). First European data from the network of severe allergic reactions (NORA). Allergy.

[7] Antonicelli L., Bilò M.B., Bonifazi F. (2002). Epidemiology of Hymenoptera allergy. Curr. Opin. Allergy Clin. Immunol..

[8] Bilò M.B., Bonifazi F. (2009). The natural history and epidemiology of insect venom allergy: Clinical implications. Clin. Exp. Allergy.

[9] Blank S., Korošec P., Slusarenko B.O., Ollert M., Hamilton R.G. (2024). Venom Component Allergen IgE Measurement in the Diagnosis and Management of Insect Sting Allergy. J. Allergy Clin. Immunol. Pract..

[10] Blank S., Haemmerle S., Jaeger T., Russkamp D., Ring J., Schmidt-Weber C.B., Ollert M. (2019). Prevalence of Hymenoptera venom allergy and sensitization in the population-representative German KORA cohort. Allergo J. Int..

[11] Golden D.B., Marsh D.G., Kagey-Sobotka A., Freidhoff L., Szklo M., Valentine M.D., Lichtenstein L.M. (1989). Epidemiology of insect venom sensitivity. JAMA.

[12] Biló B.M., Rueff F., Mosbech H., Bonifazi F., Oude-Elberink J.N. (2005). EAACI Interest Group on Insect Venom Hypersensitivity. Diagnosis of Hymenoptera venom allergy. Allergy.

[13] Sturm G.J., Kranzelbinder B., Schuster C., Sturm E.M., Bokanovic D., Vollmann J., Crailsheim K., Hemmer W., Aberer W. (2014). Sensitization to Hymenoptera venoms is common, but systemic sting reactions are rare. J. Allergy Clin. Immunol..

[14] Shade K.C., Conroy M.E., Washburn N., Kitaoka M., Huynh D.J., Laprise E., Patil S.U., Shreffler W.G., Anthony R.M. (2020). Sialylation of immunoglobulin E is a determinant of allergic pathogenicity. Nature.

[15] van de Veen W., Stanic B., Yaman G., Wawrzyniak M., Söllner S., Akdis D.G., Rückert B., Akdis C.A., Akdis M. (2013). IgG4 production is confined to human IL-10-producing regulatory B cells that suppress antigen-specific immune responses. J. Allergy Clin. Immunol..

[16] Worm M., Francuzik W., Renaudin J.M., Bilo M.B., Cardona V., Scherer Hofmeier K., Köhli A., Bauer A., Christoff G., Cichocka-Jarosz E. (2018). Factors increasing the risk for a severe reaction in anaphylaxis: An analysis of data from The European Anaphylaxis Registry. Allergy.

[17] Bonadonna P., Zanotti R., Pagani M., Bonifacio M., Scaffidi L., Olivieri E., Franchini M., Reccardini F., Costantino M.T., Roncallo C. (2018). Anaphylactic Reactions After Discontinuation of Hymenoptera Venom Immunotherapy: A Clonal Mast Cell Disorder Should Be Suspected. J. Allergy Clin. Immunol. Pract..

[18] Boggs N.A., Tanasi I., Hartmann K., Zanotti R., Gonzalez-de-Olano D. (2024). Mast Cell Disorders and Hymenoptera Venom-Triggered Anaphylaxis: Evaluation and Management. J. Allergy Clin. Immunol. Pract..

[19] Alvarez-Twose I., Bonadonna P., Matito A., Zanotti R., González-de-Olano D., Sánchez-Muñoz L., Morgado J.M., Orfao A., Escribano L. (2013). Systemic mastocytosis as a risk factor for severe Hymenoptera sting-induced anaphylaxis. J. Allergy Clin. Immunol..

[20] Golden D.B.K., Wang J., Waserman S., Akin C., Campbell R.L., Ellis A.K., Greenhawt M., Lang D.M., Ledford D.K., Lieberman J. (2024). Anaphylaxis: A 2023 practice parameter update. Ann. Allergy Asthma Immunol..

[21] Mingomataj E.Ç., Bakiri A.H., Ibranji A., Sturm G.J. (2014). Unusual reactions to hymenoptera stings: What should we keep in mind?. Clin. Rev. Allergy Immunol..

[22] Reisman R.E. (2005). Unusual reactions to insect stings. Curr. Opin. Allergy Clin. Immunol..

[23] Castagnoli R., Giovannini M., Mori F., Barni S., Pecoraro L., Arasi S., Saretta F., Mastrorilli C., Liotti L., Caminiti L. (2021). Unusual Reactions to Hymenoptera Stings: Current Knowledge and Unmet Needs in the Pediatric Population. Front. Med..

[24] Blank S., Jakwerth C.A., Zissler U.M., Schmidt-Weber C.B. (2022). Molecular determination of insect venom allergies. Expert Rev. Mol. Diagn..

[25] Bilò M.B., Cinti B., Brianzoni M.F., Braschi M.C., Bonifazi M., Antonicelli L. (2012). Honeybee venom immunotherapy: A comparative study using purified and nonpurified aqueous extracts in patients with normal Basal serum tryptase concentrations. J. Allergy.

[26] Moreno M., Giralt E. (2015). Three valuable peptides from bee and wasp venoms for therapeutic and biotechnological use: Melittin, apamin and mastoparan. Toxins.

[27] Fitzgerald K.T., Flood A.A. (2006). Hymenoptera stings. Clin. Tech. Small Anim. Pract..

[28] Rostaher A., Fischer N.M., Vigani A., Steblaj B., Martini F., Brem S., Favrot C., Kosnik M. (2023). Hymenoptera Venom Immunotherapy in Dogs: Safety and Clinical Efficacy. Animals.

[29] Haight K.L., Tschinkel W.R. (2003). Patterns of venom synthesis and use in the fire ant, Solenopsis invicta. Toxicon.

[30] Fernández J. (2004). Distribution of vespid species in Europe. Curr. Opin. Allergy Clin. Immunol..

[31] Korošec P., Jakob T., Harb H., Heddle R., Karabus S., de Lima Zollner R., Selb J., Thong B.Y., Zaitoun F., Golden D.B.K. (2019). Worldwide perspectives on venom allergy. World Allergy Organ. J..

[32] Menchetti M., Schifani E., Alicata A., Cardador L., Sbrega E., Toro-Delgado E., Vila R. (2023). The invasive ant Solenopsis invicta is established in Europe. Curr. Biol..

[33] Schifani E., Grunicke D., Montechiarini A., Pradera C., Vila R., Menchetti M. (2024). Alien ants spreading through Europe: *Brachyponera chinensis* and *Nylanderia vividula* in Italy. Biodivers. Data J..

[34] Fernández-Meléndez S., Miranda A., García-González J.J., Barber D., Lombardero M. (2007). Anaphylaxis caused by imported red fire ant stings in Málaga, Spain. J. Investig. Allergol. Clin. Immunol..

[35] Lee E.K., Jeong K.Y., Lyu D.P., Lee Y.W., Sohn J.H., Lim K.J., Hong C.S., Park J.W. (2009). Characterization of the major allergens of *Pachycondyla chinensis* in ant sting anaphylaxis patients. Clin. Exp. Allergy.

[36] Wanandy T., Gueven N., Davies N.W., Brown S.G., Wiese M.D. (2015). Pilosulins: A review of the structure and mode of action of venom peptides from an Australian ant *Myrmecia pilosula*. Toxicon.

[37] Li R., Zhang L., Fang Y., Han B., Lu X., Zhou T., Feng M., Li J. (2013). Proteome and phosphoproteome analysis of honeybee (*Apis mellifera*) venom collected from electrical stimulation and manual extraction of the venom gland. BMC Genom..

[38] Grossi V., Severino M., Massolo A., Infantino M., Laureti F., Macchia D., Meucci E., Francescato E., Pantera B., Ebbli A. (2023). *Vespa velutina nigrithorax* venom allergy: Inhibition studies approach for the choice of specific immunotherapy. Eur. Ann. Allergy Clin. Immunol..

[39] Pantera B., Hoffman D.R., Carresi L., Cappugi G., Turillazzi S., Manao G., Severino M., Spadolini I., Orsomando G., Moneti G. (2003). Characterization of the major allergens purified from the venom of the paper wasp *Polistes gallicus*. Biochim. Biophys. Acta..

[40] de Graaf D.C., Aerts M., Danneels E., Devreese B. (2009). Bee, wasp and ant venomics pave the way for a component-resolved diagnosis of sting allergy. J. Proteom..

[41] Hilger C., Villaseñor A., Hoffmann-Sommergruber K., Santos A., de la Vecillas L., Dramburg S., Blank S., Kuehn A., Giovannini M., Castagnoli R. (2024). Molecular Allergology Pocket Guide.

[42] Elieh Ali Komi D., Shafaghat F., Zwiener R.D. (2018). Immunology of Bee Venom. Clin. Rev. Allergy Immunol..

[43] King T.P., Wittkowski K.M. (2011). Hyaluronidase and hyaluronan in insect venom allergy. Int. Arch. Allergy Immunol..

[44] Spillner E., Blank S., Jakob T. (2014). Hymenoptera allergens: From venom to “venome”. Front. Immunol..

[45] Jovanovic D., Peric-Popadic A., Djuric V., Stojanovic M., Lekic B., Milicevic O., Bonaci-Nikolic B. (2023). Molecular diagnostics and inhibition of cross-reactive carbohydrate determinants in Hymenoptera venom allergy. Clin. Transl. Allergy.

[46] Schrautzer C., Bokanovic D., Hemmer W., Lang R., Hawranek T., Schwarz I., Aberer W., Sturm E., Sturm G.J. (2016). Sensitivity and specificity of Hymenoptera allergen components depend on the diagnostic assay employed. J. Allergy Clin. Immunol..

[47] Burzyńska M., Piasecka-Kwiatkowska D. (2021). A Review of Honeybee Venom Allergens and Allergenicity. Int. J. Mol. Sci..

[48] Köhler J., Blank S., Müller S., Bantleon F., Frick M., Huss-Marp J., Lidholm J., Spillner E., Jakob T. (2014). Component resolution reveals additional major allergens in patients with honeybee venom allergy. J. Allergy Clin. Immunol..

[49] Blank S., Seismann H., Michel Y., McIntyre M., Cifuentes L., Braren I., Grunwald T., Darsow U., Ring J., Bredehorst R. (2011). Api m 10, a genuine A. mellifera venom allergen, is clinically relevant but underrepresented in therapeutic extracts. Allergy.

[50] Ruiz B., Serrano P., Verdú M., Moreno C. (2015). Sensitization to Api m 1, Api m 2, and Api m 4: Association with safety of bee venom immunotherapy. Ann. Allergy Asthma Immunol..

[51] Hoffman D.R. (2006). Hymenoptera venom allergens. Clin. Rev. Allergy Immunol..

[52] Kopač P., Custovic A., Zidarn M., Šilar M., Šelb J., Bajrović N., Eržen R., Košnik M., Korošec P. (2021). Biomarkers of the Severity of Honeybee Sting Reactions and the Severity and Threshold of Systemic Adverse Events During Immunotherapy. J. Allergy Clin. Immunol. Pract..

[53] Jakob T., Rauber M.M., Perez-Riverol A., Spillner E., Blank S. (2020). The Honeybee Venom Major Allergen Api m 10 (Icarapin) and Its Role in Diagnostics and Treatment of Hymenoptera Venom Allergy. Curr. Allergy Asthma Rep..

[54] Bidovec-Stojkovič U., Vachová M., Košnik Ž., Košnik M., Panzner P., Volfand J., Homšak M., Berce V., Korošec P. (2020). Methodological and diagnostic relevance of IgEs to recombinant allergens Api m 1 and Ves v 5 determined by the multiplex test ImmunoCAP ISAC. Clin. Exp. Allergy.

[55] Jakob T., Spillner E. (2017). Comparing sensitivity of Hymenoptera allergen components on different diagnostic assay systems: Comparing apples and oranges?. J. Allergy Clin. Immunol..

[56] Sturm G.J., Schrautzer C., Arzt L., Aberer W. (2017). Reply. J. Allergy Clin. Immunol..

[57] Lambert C., Birnbaum J., Dzviga C., Hutt N., Apoil P.A., Bienvenu F., Drouet M., Beauvillain C., Brabant S., Guilloux L. (2018). Antigen 5-spiked Vespula and Polistes venom extracts for Vespid allergy diagnostics: A French multicenter study. Ann. Allergy Asthma Immunol..

[58] Kukkonen A.K., Pelkonen A.S., Edelman S.M., Kauppi P.M., Mäkelä M.J. (2018). Component-resolved diagnosis in selecting patients for yellowjacket venom immunotherapy. Ann. Allergy Asthma Immunol..

[59] Zink A., Schuster B., Winkler J., Eyerich K., Darsow U., Brockow K., Eberlein B., Biedermann T. (2019). Allergy and sensitization to Hymenoptera venoms in unreferred adults with a high risk of sting exposure. World Allergy Organ. J..

[60] Bazon M.L., Silveira L.H., Simioni P.U., Brochetto-Braga M.R. (2018). Current Advances in Immunological Studies on the Vespidae Venom Antigen 5: Therapeutic and Prophylaxis to Hypersensitivity Responses. Toxins.

[61] Caruso B., Bonadonna P., Bovo C., Melloni N., Lombardo C., Senna G., Lippi G. (2016). Wasp venom allergy screening with recombinant allergen testing. Diagnostic performance of rPol d 5 and rVes v 5 for differentiating sensitization to Vespula and Polistes subspecies. Clin. Chim. Acta.

[62] Whyte A.F., Popescu F.D., Carlson J. (2020). Tabanidae insect (horsefly and deerfly) allergy in humans: A review of the literature. Clin. Exp. Allergy.

[63] An S., Ma D., Wei J.F., Yang X., Yang H.W., Yang H., Xu X., He S., Lai R. (2011). A novel allergen Tab y 1 with inhibitory activity of platelet aggregation from salivary glands of horseflies. Allergy.

[64] Ma D., Li Y., Dong J., An S., Wang Y., Liu C., Yang X., Yang H., Xu X., Lin D. (2011). Purification and characterization of two new allergens from the salivary glands of the horsefly, *Tabanus yao*. Allergy.

[65] Bilò M.B., Martini M., Bonadonna P., Cinti B., Da Re M., Gabrielli O., Olivieri F., Salgarolo V., Zanoni G., Villalta D. (2021). Prevalence of Pol d 1 Sensitization in Polistes dominula Allergy and Its Diagnostic Role in Vespid Double-Positivity. J. Allergy Clin. Immunol. Pract..

[66] Blank S., Bazon M.L., Grosch J., Schmidt-Weber C.B., Brochetto-Braga M.R., Bilò M.B., Jakob T. (2020). Antigen 5 Allergens of Hymenoptera Venoms and Their Role in Diagnosis and Therapy of Venom Allergy. Curr. Allergy Asthma Rep..

[67] Monsalve R.I., Vega A., Marqués L., Miranda A., Fernández J., Soriano V., Cruz S., Domínguez-Noche C., Sánchez-Morillas L., Armisen-Gil M. (2012). Component-resolved diagnosis of vespid venom-allergic individuals: Phospholipases and antigen 5s are necessary to identify Vespula or Polistes sensitization. Allergy.

[68] Schiener M., Eberlein B., Moreno-Aguilar C., Pietsch G., Serrano P., McIntyre M., Schwarze L., Russkamp D., Biedermann T., Spillner E. (2017). Application of recombinant antigen 5 allergens from seven allergy-relevant Hymenoptera species in diagnostics. Allergy.

[69] Savi E., Peveri S., Makri E., Pravettoni V., Incorvaia C. (2016). Comparing the ability of molecular diagnosis and CAP-inhibition in identifying the really causative venom in patients with positive tests to Vespula and Polistes species. Clin. Mol. Allergy.

[70] Monsalve R.I., Gutiérrez R., Hoof I., Lombardero M. (2020). Purification and molecular characterization of phospholipase, antigen 5 and hyaluronidases from the venom of the Asian hornet (*Vespa velutina*). PLoS ONE.

[71] Quercia O., Cova V., Martini M., Cortellini G., Murzilli F., Bignardi D., Cilia M., Scarpa A., Bilò M.B. (2018). CAP-Inhibition, Molecular Diagnostics, and Total IgE in the Evaluation of Polistes and Vespula Double Sensitization. Int. Arch. Allergy Immunol..

[72] Potiwat R., Sitcharungsi R. (2015). Ant allergens and hypersensitivity reactions in response to ant stings. Asian Pac. J. Allergy Immunol..

[73] Jeong K.Y., Yi M.H., Son M., Lyu D., Lee J.H., Yong T.S., Park J.W. (2016). IgE Reactivity of Recombinant Pac c 3 from the Asian Needle Ant (*Pachycondyla chinensis*). Int. Arch. Allergy Immunol..

[74] Popescu F.D., Vieru M. (2018). Precision medicine allergy immunoassay methods for assessing immunoglobulin E sensitization to aeroallergen molecules. World J. Methodol..

[75] Kleine-Tebbe J., Jakob T. (2015). Molecular allergy diagnostics using IgE singleplex determinations: Methodological and practical considerations for use in clinical routine: Part 18 of the Series Molecular Allergology. Allergo J. Int..

[76] Neis M.M., Merk H.F. (2012). Value of component-based diagnostics in IgE-mediated hymenoptera sting reactions. Cutan. Ocul. Toxicol..

[77] Thermo Fisher Scientific Immunodiagnostics Product Catalog 2024. www.abacusdx.com/media/PU_ProductCatalogue_2024.pdf.

[78] Watanabe M., Hirata H., Arima M., Hayashi Y., Chibana K., Yoshida N., Ikeno Y., Fukushima Y., Komura R., Okazaki K. (2012). Measurement of Hymenoptera venom specific IgE by the IMMULITE 3gAllergy in subjects with negative or positive results by ImmunoCAP. Asia Pac. Allergy..

[79] Siemens Healthcare Allergy Menu. https://marketing.webassets.siemens-healthineers.com/667ff707799b2896/6da8b9913b44/30-21-DX-999-76_IMMULITE-Allergy_Menu_FINAL.pdf.

[80] Pfender N., Lucassen R., Offermann N., Schulte-Pelkum J., Fooke M., Jakob T. (2012). Evaluation of a Novel Rapid Test System for the Detection of Specific IgE to Hymenoptera Venoms. J. Allergy.

[81] Cabrera C.M., Palacios-Cañas A., Joyanes-Romo J.B., Urra J.M., Mur P. (2022). Basophil activation test as alternative method to CAP-inhibition in patients with double sensitization to vespid venoms. Mol. Immunol..

[82] Caruso B., Bonadonna P., Severino M.G., Manfredi M., Dama A., Schiappoli M., Rizzotti P., Senna G., Passalacqua G. (2007). Evaluation of the IgE cross-reactions among vespid venoms. A possible approach for the choice of immunotherapy. Allergy.

[83] Straumann F., Bucher C., Wüthrich B. (2000). Double sensitization to honeybee and wasp venom: Immunotherapy with one or with both venoms? Value of FEIA inhibition for the identification of the cross-reacting ige antibodies in double-sensitized patients to honeybee and wasp venom. Int. Arch. Allergy Immunol..

[84] Euroimmun Euroline Allergy—Efficient Multiparameter Profiles. www.euroimmun.com/documents/Indications/Allergology/Multiplex-immunoblots/Euroline/DP_3000_I_UK_D.pdf.

[85] Euroimmun Insect Venoms. www.euroimmun.com/products/allergy-diagnostics/id/insect-venoms/.

[86] Macro Array Diagnostics ALEX2® Allergen List. https://macroarraydx.com.ua/downloads/alex2_allergen_list_en.pdf.

[87] Bemanian M.H., Shokouhi Shoormasti R., Arshi S., Jafari M., Shokri S., Fallahpour M., Nabavi M., Zaremehrjardi F. (2024). The role of molecular diagnosis in anaphylactic patients with dual or triple-sensitization to Hymenoptera venoms. Allergy Asthma Clin. Immunol..

[88] Tuppo L., Giangrieco I., Alessandri C., Ricciardi T., Rafaiani C., Ciancamerla M., Ferrara R., Zennaro D., Bernardi M.L., Tamburrini M. (2018). Pomegranate chitinase III: Identification of a new allergen and analysis of sensitization patterns to chitinases. Mol. Immunol..

[89] Bonadonna P., Korosec P., Nalin F., Golden D.B.K. (2023). Venom Anaphylaxis: Decision Points for a More Aggressive Workup. J. Allergy Clin. Immunol. Pract..

[90] Schmidle P., Blank S., Altrichter S., Hoetzenecker W., Brockow K., Darsow U., Biedermann T., Eberlein B. (2023). Basophil Activation Test in Double-Sensitized Patients with Hymenoptera Venom Allergy: Additional Benefit of Component-Resolved Diagnostics. J. Allergy Clin. Immunol. Pract..

[91] Waldherr S., Hils M., Köberle M., Brockow K., Darsow U., Blank S., Biedermann T., Eberlein B. (2024). Basophil activation in insect venom allergy: Comparison of an established test using liquid reagents with a test using 5-color tubes with dried antibody reagents. BMC Immunol..

[92] Eberlein-König B., Varga R., Mempel M., Darsow U., Behrendt H., Ring J. (2006). Comparison of basophil activation tests using CD63 or CD203c expression in patients with insect venom allergy. Allergy.

[93] Depince-Berger A.E., Sidi-Yahya K., Jeraiby M., Lambert C. (2017). Basophil activation test: Implementation and standardization be-tween systems and between instruments. Cytom. Part A..

[94] Eberlein B., Brockow K., Darsow U., Biedermann T., Blank S. (2024). Basophil activation test in Hymenoptera venom allergy. Allergol. Select.

[95] Eberlein B. (2020). Basophil Activation as Marker of Clinically Relevant Allergy and Therapy Outcome. Front. Immunol..

[96] Hemmings O., Kwok M., McKendry R., Santos A.F. (2018). Basophil Activation Test: Old and New Applications in Allergy. Curr. Allergy Asthma Rep..

[97] Korošec P., Šilar M., Eržen R., Čelesnik N., Bajrović N., Zidarn M., Košnik M. (2013). Clinical routine utility of basophil activation testing for diagnosis of hymenoptera-allergic patients with emphasis on individuals with negative venom-specific IgE antibodies. Int. Arch. Allergy Immunol..

[98] Hoffmann H.J., Santos A.F., Mayorga C., Nopp A., Eberlein B., Ferrer M., Rouzaire P., Ebo D.G., Sabato V., Sanz M.L. (2015). The clinical utility of basophil activation testing in diagnosis and monitoring of allergic disease. Allergy.

[99] Buhlmann CAST® Allergen List. www.buhlmannlabs.ch/wp-content/uploads/2023/01/Allergen-List-LA014ML-27-E.pdf.

[100] Balzer L., Pennino D., Blank S., Seismann H., Darsow U., Schnedler M., McIntyre M., Ollert M.W., Durham S.R., Spillner E. (2014). Basophil activation test using recombinant allergens: Highly specific diagnostic method complementing routine tests in wasp venom allergy. PLoS ONE.

[101] Sturm G.J., Biló M.B., Bonadonna P., Hemmer W., Caruso B., Bokanovic D., Aberer W. (2012). Ves v 5 can establish the diagnosis in patients without detectable specific IgE to wasp venom and a possible north-south difference in Api m 1 sensitization in Europe. J. Allergy Clin. Immunol..

[102] Grosch J., Eberlein B., Waldherr S., Pascal M., San Bartolomé C., De La Roca Pinzón F., Dittmar M., Hilger C., Ollert M., Biedermann T. (2021). Characterization of New Allergens from the Venom of the European Paper Wasp *Polistes dominula*. Toxins.

[103] Schiener M., Hilger C., Eberlein B., Pascal M., Kuehn A., Revets D., Planchon S., Pietsch G., Serrano P., Moreno-Aguilar C. (2018). The high molecular weight dipeptidyl peptidase IV Pol d 3 is a major allergen of Polistes dominula venom. Sci. Rep..

[104] Urra J.M., Pérez-Lucendo I., Extremera A., Feo-Brito F., Alfaya T. (2020). The Method for Selecting Basophils Might Be Determinant in the Basophil Activation Test in Patients with Mastocytosis. J. Investig. Allergol. Clin. Immunol..

[105] Michalet X., Pinaud F.F., Bentolila L.A., Tsay J.M., Doose S., Li J.J., Sundaresan G., Wu A.M., Gambhir S.S., Weiss S. (2005). Quantum dots for live cells, in vivo imaging, and diagnostics. Science.

[106] Koren A., Lunder M., Molek P., Kopač P., Zahirović A., Gattinger P., Mittermann I., Valenta R., Korošec P. (2020). Fluorescent labeling of major honeybee allergens Api m 1 and Api m 2 with quantum dots and the development of a multiplex basophil activation test. Allergy.

[107] Ruëff F., Bauer A., Becker S., Brehler R., Brockow K., Chaker A.M., Darsow U., Fischer J., Fuchs T., Gerstlauer M. (2023). Diagnosis and treatment of Hymenoptera venom allergy: S2k Guideline of the German Society of Allergology and Clinical Immunology (DGAKI) in collaboration with the Arbeitsgemeinschaft für Berufs- und Umweltdermatologie e.V. (ABD), the Medical Association of German Allergologists (AeDA), the German Society of Dermatology (DDG), the German Society of Oto-Rhino-Laryngology, Head and Neck Surgery (DGHNOKC), the German Society of Pediatrics and Adolescent Medicine (DGKJ), the Society for Pediatric Allergy and Environmental Medicine (GPA), German Respiratory Society (DGP), and the Austrian Society for Allergy and Immunology (ÖGAI). Allergol. Select.

[108] Scherer K., Bircher A.J., Heijnen I.A. (2009). Diagnosis of stinging insect allergy: Utility of cellular in-vitro tests. Curr. Opin. Allergy Clin. Immunol..

[109] Sainte-Laudy J., Sabbah A., Drouet M., Lauret M.G., Loiry M. (2000). Diagnosis of venom allergy by flow cytometry. Correlation with clinical history, skin tests, specific IgE, histamine and leukotriene C4 release. Clin. Exp. Allergy.

[110] Steering Committee Authors, Review Panel Members (2020). A WAO-ARIA-GA2LEN consensus document on molecular-based allergy diagnosis (PAMD@): Update 2020. World Allergy Organ. J..

[111] Kleine-Tebbe J., Jappe U. (2017). Molecular allergy diagnostic tests: Development and relevance in clinical practice. Allergol. Select..

[112] Tischler S., Trautmann A., Goebeler M., Stoevesandt J. (2024). Bee/Vespula Venom-Specific IgE Ratio Greater Than 5:1 Indicates Culprit Insect in Double-Sensitized Patients. J. Allergy Clin. Immunol. Pract..

[113] Carballada González F., Abel-Fernández E., González Guzmán L.A., Pineda de la Losa F. (2024). Component-Based Assessment of the Main Allergens in Honeybee Venom in a Spanish Allergic Population. J. Investig. Allergol. Clin. Immunol..

[114] Valles S.M., Strong C.A., Callcott A.M. (2016). Development of a lateral flow immunoassay for rapid field detection of the red imported fire ant, Solenopsis invicta (Hymenoptera: Formicidae). Anal. Bioanal. Chem..

[115] Giraldo-Tugores M., Vaquero-Rey A., Santacruz-Santos M., Rodríguez-Martín E., De Andrés A., Ballester-Gonzalez R., Barra-Castro A., Fernández-Lozano C., Martinez-Botas J., Antolín-Amérigo D. (2023). Application of In Vitro Tests to Establish an Accurate Diagnosis of Double Sensitization to Vespula and Polistes Species. J. Investig. Allergol. Clin. Immunol..

[116] Ruiz-Leon B., Serrano P., Vidal C., Moreno-Aguilar C. (2022). Management of Double Sensitization to Vespids in Europe. Toxins.

[117] Eberlein B., Krischan L., Darsow U., Ollert M., Ring J. (2012). Double positivity to bee and wasp venom: Improved diagnostic procedure by recombinant allergen-based IgE testing and basophil activation test including data about cross-reactive carbohydrate determinants. J. Allergy Clin. Immunol..

[118] Sturm G.J., Jin C., Kranzelbinder B., Hemmer W., Sturm E.M., Griesbacher A., Heinemann A., Vollmann J., Altmann F., Crailsheim K. (2011). Inconsistent results of diagnostic tools hamper the differentiation between bee and vespid venom allergy. PLoS ONE.

[119] Grosch J., Eberlein B., Waldherr S., Pascal M., Dorn B., San Bartolomé C., De La Roca Pinzón F., Schiener M., Darsow U., Biedermann T. (2024). Comparative Assessment of the Allergenicity of Hyaluronidases from Polistes dominula (Pol d 2), Vespula vulgaris (Ves v 2), and Apis mellifera Venom (Api m 2). Toxins.

[120] Sturm G.J., Varga E.M., Roberts G., Mosbech H., Bilò M.B., Akdis C.A., Antolín-Amérigo D., Cichocka-Jarosz E., Gawlik R., Jakob T. (2018). EAACI guidelines on allergen immunotherapy: Hymenoptera venom allergy. Allergy.

[121] Bertlich M., Weber F., Bertlich I., Kendziora B., Rueff F., Spiegel J.L., French L.E., Oppel E. (2024). Characteristics of patients with anaphylaxis to European hornet (Vespa crabro) venom compared to anaphylaxis to wasp (Vespula spp.) venom in southern Germany. Int. Arch. Allergy Immunol..

[122] Sturm G.J., Boni E., Antolín-Amérigo D., Bilò M.B., Breynaert C., Fassio F., Spriggs K., Vega A., Ricciardi L., Arzt-Gradwohl L. (2023). Allergy to stings and bites from rare or locally important arthropods: Worldwide distribution, available diagnostics and treatment. Allergy.

[123] Treudler R., Worm M., Bauer A., Dickel H., Heine G., Jappe U., Klimek L., Raulf M., Wedi B., Wieczorek D. (2024). Occupational anaphylaxis: A Position Paper of the German Society of Allergology and Clinical Immunology (DGAKI). Allergol. Select..

[124] Frick M., Fischer J., Helbling A., Ruëff F., Wieczorek D., Ollert M., Pfützner W., Müller S., Huss-Marp J., Dorn B. (2016). Predominant Api m 10 sensitization as risk factor for treatment failure in honey bee venom immunotherapy. J. Allergy Clin. Immunol..

[125] Ruiz-León B., Navas A., Serrano P., Espinazo M., Labrador-Horrillo M., Monsalve R.I., Jurado A., Moreno-Aguilar C. (2020). Successful Adaptation of Bee Venom Immunotherapy in a Patient Monosensitized to Api m 10. J. Investig. Allergol. Clin. Immunol..

